# Population genome-wide analysis reveals historical divergence and adaptive signals in *Rubroshorea leprosula* (Dipterocarpaceae), a near-threatened tropical forest tree

**DOI:** 10.1186/s12864-026-12992-5

**Published:** 2026-05-29

**Authors:** Alias Nur-Nabilah, Kevin Kit Siong Ng, Soon Leong Lee, Zeti-Azura Mohamed-Hussein, Masaki J Kobayashi, Chin Hong Ng, Lee Hong Tnah, Chai Ting Lee, Khairuddin Perdan, Robert C. Ong, Bibian Diway, Iskandar Z Siregar, Sapto Indrioko

**Affiliations:** 1 Forestry Biotechnology Division, Forest Research Institute Malaysia (FRIM), Kepong, Selangor 52109 Malaysia; 2 Department of Applied Physics, Faculty of Science and Technology (FST), Universiti Kebangsaan Malaysia, Bangi, Selangor 43600 UKM Malaysia; 3 UKM Medical Molecular Biology Institute (UMBI), Jalan Ya’acob Latiff, Bandar Tun Razak, Kuala Lumpur, 56000 Malaysia; 4 Forestry Division, Japan International Research Center for Agricultural Sciences, Tsukuba, Ibaraki 305-8686 Japan; 5 Forest Management Division, Forestry Department of Peninsular Malaysia, Jalan Sultan Salahuddin, Kuala Lumpur, 50660 Malaysia; 6 Forest Research Centre, Sabah Forestry Department, Sandakan, Sabah 90715 Malaysia; 7 Research and Development Division, Forest Department Sarawak, Jalan Datuk Amar Kalung Ningkan, Km10, Kuching, Sarawak 93250 Malaysia; 8 Faculty of Forestry, IPB University, Bogor, 16680 Indonesia; 9 Faculty of Forestry, Gadjah Mada University, Yogyakarta, 55281 Indonesia

**Keywords:** Adaptation, Demographic history, *F*_ST_, Gene flow, Genome-environment association (GEA), Pleistocene, Whole-genome resequencing

## Abstract

**Background:**

Lowland dipterocarp forests in Southeast Asia represent one of the world’s most significant biodiversity hotspots, yet they have experienced extensive fragmentation by rapid land-use change. *Rubroshorea leprosula*, a near-threatened and economically important dipterocarp, occurs widely across Peninsular Malaysia, Sumatra and Borneo, yet its evolutionary history and adaptive potential remain poorly understood. Understanding how and why genetic variation is structured across this geographic range is essential for predicting the resilience of tropical tree species, particularly where geographical isolation and heterogeneous environments may drive localized adaptation.

**Results:**

Whole-genome resequencing of 194 individuals from 37 natural populations revealed moderate genome-wide diversity, with notable regional contrasts. Populations from Peninsular Malaysia and Sumatra exhibited higher heterozygosity and shared ancestral variation, while Bornean populations showed signals of recent demographic expansion. Population structure analyses identified two major genetic clusters corresponding to Western (Peninsular Malaysia + Sumatra) and Eastern (Borneo) lineages, with a small number of admixed individuals in northern Peninsular Malaysia suggesting historical gene flow. Demographic modelling supports divergence during the Mid- to Late-Pleistocene followed by low, asymmetric post-divergence gene flow, consistent with secondary contact during post-glacial forest reconnection across Sundaland. Integration of outlier detection (*F*_ST_ and pcadapt) with environmental association analyses (RDA and LFMM2) identified candidate loci associated with stress response, signalling and metabolic pathways, suggesting that environmental heterogeneity across the species’ range has contributed to adaptive genomic differentiation.

**Conclusions:**

This study reveals strong west-east genetic divergence in *R. leprosula* and identifies climate-associated loci linked to adaptation across temperature, elevation and precipitation gradients. These genome-wide patterns clarify evolutionary processes and provide a foundation for climate-informed conservation management of threatened dipterocarp forests.

**Supplementary Information:**

The online version contains supplementary material available at 10.1186/s12864-026-12992-5.

## Background

Southeast Asia’s lowland rainforests, dominated by Dipterocarpaceae, are among the most biodiverse and carbon-dense ecosystems globally, yet they are increasingly threatened by deforestation, habitat fragmentation, land-use change and unsustainable logging [1]. These dipterocarp forests not only act as major carbon reservoirs but also provide important ecosystem services and support major timber industries, making their conservation both an ecological and economic priority. Phylogenomic and paleobiogeographic evidence suggests that Dipterocarpaceae likely originated in tropical Africa during the mid-Cretaceous (93.5-112.2 million years ago (MYA)) and later expanded eastward via India to Southeast Asia, where they underwent rapid diversification and became structural foundation species that support the integrity and functioning of rainforest ecosystems [2–4]. Genomic work further reveals ancient whole-genome duplication events preceded this diversification [5], while more recent analyses reveal population declines likely associated with anthropogenic pressures [6].

*Rubroshorea leprosula* Miq. (synonym *Shorea leprosula*) is one of the most widespread and commercially valuable dipterocarps across Peninsular Malaysia, Sumatra and Borneo [7, 8]. As an emergent canopy species, it plays a crucial role in shaping forest structure, regeneration dynamics and ecosystem productivity, while its durable timber supports local livelihoods and regional economies. Yet, like many dipterocarps, *R. leprosula* is increasingly threatened by extensive habitat loss, climate change and disruptions to supra-annual mass flowering events. These pressures can constrain pollen and seed dispersal, thereby limiting gene flow and effective population size, and ultimately increasing the risks of inbreeding and genetic erosion [9, 10]. Genomic studies in related taxa highlight the severity of such risks. For instance, *Hopea hainanensis* and *H. reticulata* exhibit extremely low genetic diversity and elevated inbreeding in small and isolated populations [6], while *Vatica guangxiensis* revealed pronounced genetic erosion associated with extremely small population sizes [10, 11]. These findings collectively demonstrate the power of genome-wide approaches in detecting cryptic genetic erosion and adaptive constraints.

The contemporary distribution of *R. leprosula* is closely linked to the geological and climatic history of Sundaland, a major biogeographic region encompassing the Peninsular Malaysia and the islands of Sumatra and Borneo. The region is defined by a humid tropical climate with high annual rainfall and stable temperatures, conditions that sustain extensive lowland dipterocarp forests [12, 13]. However, this landscape is not uniform; significant environmental heterogeneity and distinct geographic barriers drive spatial variation in ecological conditions across the landscape [14, 15]. Central to this history is the Sunda Shelf, a shallow continental platform, which was repeatedly exposed during Pleistocene glacial periods when sea levels dropped by up to ~ 120 m, forming land bridges that connected currently isolated landmasses [16, 17]. These connections facilitated range expansion and gene flow, whereas subsequently sea levels rise during interglacial periods submerged the shelf, isolating populations on their respective landmasses. This repeated cycle of connectivity and fragmentation served as a primary driver of genetic differentiation and spatial structure in many Southeast Asian forest species today [18, 19].

Earlier studies on *R. leprosula* relied primarily on simple sequence repeats (SSRs), chloroplast DNA (cpDNA) and other low-resolution markers including allozymes, amplified fragment length polymorphism (AFLP) and random amplified polymorphic DNA (RAPD) [20–27]. While these approaches provided valuable baseline information on genetic variation and population differentiation, their limited genomic coverage restricted the resolution of genome-wide diversity, detailed demographic histories and signals of local adaptation. Advances in high-throughput next-generation sequencing (NGS) technologies and the availability of reference genomes now enable whole-genome resequencing in non-model tropical trees, facilitating the discovery of high-density single nucleotide polymorphisms (SNPs) across the genome [5, 6]. Such genome-wide data offer more robust inference of population structure, demographic history and detect adaptive variation [6, 28]. In parallel, integrated genomic approaches that combine *F*_ST_-based genome scans with genome-environment association (GEA) analyses have emerged as effective tools for identifying loci potentially under selection.

For instance, adaptive variants associated with drought tolerance have been identified in *Corymbia calophylla* [29] while, environmental associations with temperature and moisture gradients have been reported in *Castanopsis hainanensis* [30] and climate-associated SNPs in *Eucalyptus globulus* [31]. These studies indicate that adaptive variation in long-lived trees if often associated with stress-response and regulatory pathways, which may underlie adaptation in *R. leprosula*.

Understanding how genetic variation is partitioned across landscapes, how past climatic oscillations have shaped population demography and how species respond to environmental heterogeneity is crucial for predicting the resilience of dipterocarps under ongoing climate change. Despite increasing genomic insights in tropical trees, no dipterocarp species has yet been investigated at the genome-wide population scale across its full geographic range by integrating demographic modelling and GEA analyses. As a result, the relative roles of historical processes and contemporary environmental pressures in shaping dipterocarp evolutionary trajectories remain poorly understood. Here, we address this critical knowledge gap by (1) assessing genetic diversity within and among populations, (2) characterizing population structure and genomic differentiation, (3) reconstructing demographic history in relation to Pleistocene sea-level changes, and (4) identifying candidate loci associated with local adaptation using both differentiation- and environment-based genomic approaches. By integrating genomic and environmental data across Peninsular Malaysia, Sumatra and Borneo, this study presents the first genome-wide assessment demographic history and adaptive landscape of *R. leprosula*, providing insights into its evolutionary resilience and offering a robust genomic foundation to support conservation and sustainable management of Southeast Asia’s rapidly declining dipterocarp forests.

## Methods

### Population sampling and whole-genome resequencing

A total of 194 individuals of *R. leprosula* were sampled from 37 natural populations across Southeast Asia, comprising 28 populations from Peninsular Malaysia, seven from Borneo (three from Sabah, and two each from Sarawak and Kalimantan) and two from Sumatra. Formal species identification was carried out by a botanist (Mr. Kamaruddin Salleh), and total of 21 available voucher specimens were deposited at the Kepong Herbarium (KEP), Forest Research Institute Malaysia (FRIM) (Table 1). All sampling locations were recorded using a Global Positioning System (GPS) and visualized using Google Earth Pro version 7.3 (Google LLC).

**Table 1:** Geographical areas, sample size (*N*), herbarium voucher specimen numbers and access permits for 37 natural populations of *Rubroshorea leprosula* from Peninsular Malaysia, Borneo and Sumatra

No.	Region	^a^ Population	Code	Latitude	Longitude	*N*	^b^ Voucher No.	Permit No.
1	Peninsular Malaysia	Air Hitam FR	AHi	2.04838	102.77435	5	nil	PHDJU 21/2019
2		Labis FR	Lab	2.34681	103.15914	3	nil	
3		Endau Rompin SP	ERo	2.53479	103.37729	5	FRI 111,715	Penyelidikan/INV/2019/00032
4		Mersing FR	Mer	2.34247	103.63694	6	nil	CJB 012037
5		Bukit Senggeh FR	BSe	2.40398	102.45575	5	FRI 111,721	M J-PM-10-2019
6		Ayer Keroh FR	AKe	2.28337	102.30115	6	nil	
7		Kenaboi FR	Ken	3.07017	102.13643	5	FRI 111,707	46/2018
8		Pasoh FR	Ps	2.99364	102.32272	5	FRI 111,711	47/2018
9		Sungai Menyala FR	SMe	2.49857	101.88760	6	FRI 111,719	48/2018
10		Gunung Angsi FR	GAn	2.72516	102.05612	6	FRI 111,718	
11		Ulu Gombak FR	UGo	3.31140	101.70010	5	FRI 111,717	US/06/2015
12		Sungai Lalang FR	SLa	3.09083	101.87953	6	nil	
13		Korbu FR	Kor	4.88693	101.29481	5	FRI 111,720	PHD.KK.02/2019
14		Ulu Kenas FR	UKe	4.68981	100.89625	6	nil	
15		Royal Belum SP	RBe	5.63040	101.40138	4	FRI 111,706	PTNPk/PERMIT/1282/2016
16		Temenggor FR	Tem	5.52567	101.60468	5	FRI 111,701	PMH/129/2019
17		Gunung Inas FR	GIn	5.50258	100.78000	5	FRI 111,702	KU.12/2018
18		Sungai Badak FR	SBa	6.46619	100.53942	5	FRI 111,708	
19		Bukit Enggang FR	BEn	5.84055	100.73063	6	nil	PM KT 195/2018
20		Sungai Betis FR	SBe	4.76461	101.77117	5	nil	PHS DS-199-19
21		Cabang Tongkat FR	CTo	5.87543	102.25806	5	nil	DT(PM)-14-19
22		Ulu Sat FR	USa	5.73222	102.32856	6	nil	DT(PM)-18-22
23		Bukit Kesing FR	BKe	5.25953	102.87832	5	nil	1015/2019
24		Hulu Terengganu FR	HTe	4.96598	102.95417	6	FRI 111,712	
25		Beserah FR	Bes	3.82756	103.36250	5	FRI 111,709	PHDK.80/1/23Jld.9(101)
26		Jengka FR	Jen	3.73798	102.58297	5	FRI 111,710	PHD.TEM.03/2019
27		Taman Negara NP	TNe	4.40228	102.40273	3	FRI 111,716	PHD.J.37/2019
28		Lentang FR	Len	3.37562	101.99458	6	nil	PHD/BTG.100.21/10 Jld.21(4)
29	Sumatra	Bukit Tigapuluh NP	CS	-1.00000	102.50000	6	nil	DDBJ BioProject No. PRJDB8161^5^
30		Bukit Lawang NP	NS	3.50000	97.50000	6	nil	
31	Borneo	Betung Kerihun NP	WK	1.22083	113.35306	6	nil	
32		PT Sari Bumi Kusuma	LK	-1.40000	114.70000	6	nil	
33		Danum Valley CA	Dan	4.96503	117.80165	5	FRI 111,714	JKP141108019032013
34		Imbak Canyon CA	Imb	5.11882	117.04232	5	FRI 111,704	
35		Maliau Basin CA	Mal	4.74275	116.97448	5	FRI 111,705	
36		Gunung Gading NP	GGa	1.69410	109.84255	5	FRI 111,703	NCCD.907.4.4(V)-271
37		Kubah-Matang NP	KMa	1.61807	110.16455	5	FRI 111,713	

^a^*FR* Forest Reserve, *SP* State Park, *NP* National Park, *CA* Conservation Area

^b^Voucher specimens deposited to Kepong Herbarium (KEP), Forest Research Institute Malaysia (FRIM)

**Fig. 1:**
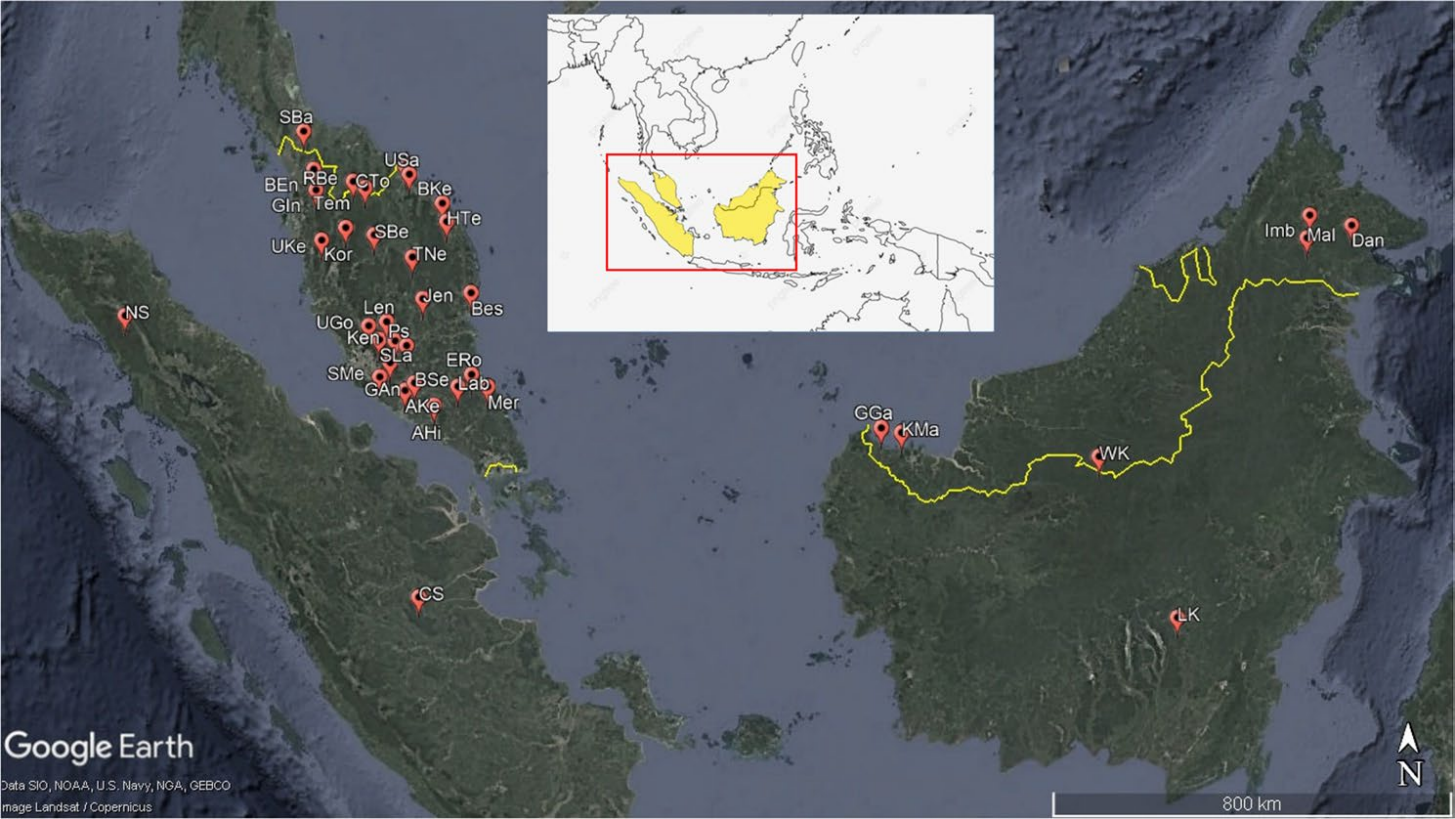
Geographical overview of 37 *Rubroshorea leprosula* populations sampled across Peninsular Malaysia, Borneo and Sumatra. Sampling locations were recorded using GPS coordinates and plotted on a map generated with Google Earth Pro (Google LLC)

Detailed information on sampling sites, sample sizes, and the corresponding access permits is provided in Table 1, and the geographic distribution of populations is shown in Fig. 1. Genomic DNA was extracted from leaves or inner bark tissues using modified cetyltrimethylammonium bromide (CTAB) protocol [32] and purified with the High Pure PCR Template Preparation Kit (Roche Diagnostics, Germany). The integrity and quality of the DNA were evaluated on 1% agarose gel electrophoresis and the concentration of the DNA samples was quantified using NanoDrop 2000 spectrophotometer (Thermo Fisher Scientific, USA). High-quality genomic DNA (2 µg per sample), with concentrations ≥ 20 ng/µl and purity ratios (A260/A280) between 1.8 and 2.0 was outsourced for whole-genome resequencing using Illumina HiSeq and NovaSeq platforms. Sequencing generated approximately 8 Gb of paired-end short reads per sample, corresponding to an average coverage depth of ~ 16×. Data for the Sumatra and Kalimantan populations were obtained from publicly available sources^5^.

### Read processing, alignment and variant calling

Raw sequencing reads were quality-checked using FASTQC v0.11.8 [33] and trimmed with Trimmomatic v0.40 [34] to remove low-quality reads and adapter sequences. Clean reads were aligned to the *R. leprosula* chromosome-scale reference genome assembly (Ng et al. unpublished data) using BWA-MEME [35]. The resulting SAM files were converted to sorted BAM format, and PCR duplicates were removed using SAMtools v1.19 [36]. Mapping statistics were summarized with MultiQC [37].

Variant calling was performed using the ‘bcftools mpileup’ and ‘bcftools call’ pipeline [36]. Initial filtering was conducted following established protocols [38, 39], with additional refinements to retain high-confidence biallelic SNPs. To minimize misalignment artifacts, all INDELs and SNPs located within 5 bp of an INDEL were excluded. Sites with low sequencing depth (DP < 5), low variant quality (QUAL < 10) and > 20% missing genotypes were further filtered.

### SNP filtering and dataset generation

To address distinct analytical requirements, three primary master datasets were generated from the filtered biallelic SNPs. First, an imputed and LD-pruned neutral dataset was developed by phasing and imputing sites with MAF > 0.05 using Beagle v5.4 [40, 41], followed by linkage disequilibrium (LD) pruning in PLINK v1.90 [42] with a threshold of squared correlation coefficient (*r*^2^ = 0.1; --indep-pairwise 50 10 0.1). Putatively adaptive outliers identified via pcadapt (*K* = 2, FDR < 0.05) [43, 44] were subsequently removed to ensure a strictly neutral signal. Second, a non-imputed, unpruned neutral dataset was generated by excluding these pcadapt outliers from the original biallelic sites while preserving observed genotypes. This approach was taken to avoid imputation-related biases, thereby, ensuring the integrity of the site frequency spectrum (SFS) and genetic diversity estimates (e.g., *H*_O_, *H*_E_, and *π*) [45–47]. This dataset was maintained in two versions using MAF > 0.05 and MAF > 0.01 thresholds to accommodate different frequency-based analyses. Finally, a non-imputed full dataset (MAF > 0.01, unpruned) was retained to provide a complete genomic profile. By including both neutral and outlier loci, this dataset enabled the identification of adaptive signals via genome-wide selection scans and genome-environment association (GEA) analyses.

### Genomic landscape and linkage disequilibrium decay

To characterize the resulting genomic landscape, genome-wide SNP density was calculated in non-overlapping 100 kb windows across the seven major scaffolds. Linkage disequilibrium (LD) decay was estimated using PopLDdecay v3.41 [48], with the decay threshold defined as the physical distance at which the average squared correlation coefficient (*r*²) dropped to 50% of its observed maximum value.

### Genetic diversity and neutrality testing

Genetic diversity and neutrality were assessed at the population level using two complementary approaches to capture both individual-specific and window-based genomic variation. First, observed (*H*_O_) and expected (*H*_E_) heterozygosity were calculated for each individual within a population using VCFtools [36], based on the non-imputed, neutral SNP dataset (MAF > 0.05).

These individual metrics were then averaged to produce population-level means, with the reported standard deviation (SD) representing inter-individual variation rather than SNP-wise variance. Second, nucleotide diversity (π), Watterson’s theta (*θ*) and Tajima’s *D* [49] were estimated in non-overlapping 100 kb windows using Pixy [50]. By incorporating both variant and invariant sites, this window-based approach explicitly accounts for missing data and avoids the biases inherent in SNP-only datasets. For these metrics, the SD represents genomic heterogeneity across windows within each population. All metrics were summarized as population-level means and standard deviations (SD), representing genomic heterogeneity across the 37 studied populations.

### Population genetic structure

Population structure was inferred from the independent, neutral SNP dataset (imputed, LD-pruned, MAF > 0.05) using three complementary approaches: Bayesian clustering, principal component analysis (PCA) and Maximum Likelihood (ML) phylogenetic reconstruction. Admixture analysis was performed using ADMIXTURE v1.3.0 [51], with the number of ancestral clusters (*K*) tested from 1 to 6. The optimal *K* was determined based on the lowest cross-validation (CV) error [52]. To ensure result stability, ten independent runs for each *K* were summarized and visualized using CLUMPAK [53]. To further explore genetic clustering and visualize the major axes of variation among individuals, PCA was conducted using PLINK v1.90 [42] and visualized with custom R scripts. To visualize the evolutionary relationships among individuals, an ML phylogenetic tree was constructed using IQ-TREE v2.4.0 [54]. The best-fit nucleotide substitution model, TVM + F+I+R4, was selected via ModelFinder [55] based on the Bayesian Information Criterion (BIC). Branch support was assessed using the Ultrafast Bootstrap (UFBoot2) approximation with 1,000 replicates [56]. The resulting tree was visualized as an unrooted phylogram using Interactive Tree of Life (iTOL) platform [57].

### Genetic differentiation and spatial analysis

Genetic differentiation among the 37 studied populations was quantified using pairwise Hudson’s *F*_ST_ estimates [58]. This estimator was selected for its robustness in handling varying sample sizes across many populations [59]. The analysis was performed in PLINK v2.0 [42, 60] using the independent, neutral SNP dataset. Pairwise *F*_ST_ values were subsequently linearized as *F*_ST_ / (1 – *F*_ST_) to facilitate spatial analysis [61, 62]. In accordance with standard population genetic interpretations, all negative pairwise *F*_ST_ estimates were adjusted to zero to represent a total absence of differentiation [63]. The magnitude of differentiation and the hierarchical relationships among all 37 populations were visualized via a clustered heatmap with a Neighbor-Joining (NJ) dendrogram, generated using the *pheatmap* package in R.

To partition genetic variation across hierarchical scales, an Analysis of Molecular Variance (AMOVA) [64] was conducted using the poppr.amova() function in the *poppr* package [65]. Total genetic variance was partitioned into components representing variation among and within populations. The statistical significance of these variance components and the associated fixation index (ΦST) was evaluated using the randtest() function in the *ade4* package [66] with 999 permutations.

Additionally, spatial genetic structure was assessed through isolation by distance (IBD) analysis. The correlation between linearized genetic distance (*F*_ST_ / (1 – *F*_ST_)) [61] and the logarithm of geographic distance (km) was evaluated using a Mantel test with 10,000 permutations in the *vegan* package [67]. Geographic distances were calculated from population GPS coordinates using the *geosphere* package. Results for all spatial and variance analyses were considered statistically significant at *p* < 0.001.

### Demographic analysis

Demographic history of *R. leprosula* was inferred using Fastsimcoal2 v2.8 [68] by fitting alternative demographic models to the folded joint site frequency spectrum (SFS). To ensure that the inferences reflected neutral evolutionary processes rather than the effects of selection, the joint SFS was generated using easySFS (https://github.com/isaacovercast/easySFS) based on the non-imputed, unpruned and neutral SNP dataset (MAF > 0.01). Utilizing unpruned data with a low MAF threshold preserved the high-resolution signal of rare variants, which is critical for capturing recent demographic events and providing a robust fit for the observed SFS. Two primary demographic scenarios were tested based on the population structure inferred at *K* = 2. Model 1 (Isolation-Only; IO) assumed that Western and Eastern clusters diverged from a common ancestral population without subsequent gene flow. In contrast, Model 2 (Isolation-with-Migration; IM) assumed divergence followed by asymmetric bidirectional migration between the two clusters. Both models estimated the effective population sizes of descendant populations (*N*_W_ and *N*_E_), ancestral population size (*N*_ANC_) and divergence time in generations (*T*_DIV_). A per-site mutation rate ranging from 1.0 × 10^− 9^ to 3.0 × 10^− 9^ was applied [26]. The IM model additionally incorporated bidirectional migration rates (*M*_WE_ and *M*_EW_) to account for post-divergence gene flow, while the IO model assumed complete isolation after divergence.

Divergence times were converted from generations to calendar years using a biologically realistic generation-time range of 30–150 years, following Ohtani et al. [26].

Each model was executed for 100 independent runs, with 100,000 coalescent simulations and 50 expectation-conditional maximization (ECM) cycles per run. The best-fitting run for each model was selected based on the maximum composite likelihood. Model selection was performed using the Akaike Information Criterion (AIC = 2*k* − 2ln*L*), where *k* is the number of parameters in the model and *L* is the maximum composite likelihood. AIC is widely used for demographic and evolutionary model comparison because it balances model fit and complexity [69–71]. Confidence intervals for the best-fit model parameters were obtained from 100 non-parametric bootstrap replicates of the observed SFS [72].

### Identification of candidate loci for local adaptation

To identify genomic regions associated with local adaptation, we integrated differentiation-based selection scans with genome-environment association (GEA) analyses. This two-step approach was designed to capture high-confidence loci that exhibit both strong lineage divergence and significant correlation with environmental variables. First, a genome-wide scan was performed to detect loci under putatively divergent selection between the Western and Eastern clusters. Pairwise *F*_ST_ values for all SNPs were calculated using VCFtools based on the non-imputed full dataset (MAF > 0.01, unpruned) to maintain maximum genomic resolution.

SNPs exceeding the 99^th^ percentile of the empirical *F*_ST_ distribution were identified as putative outlier loci [73]. This non-parametric approach is robust for detecting highly differentiated markers while minimizing assumptions regarding demographic history [74, 75]. Simultaneously, pcadapt was performed on the LD-pruned dataset (MAF > 0.05) to identify outliers with FDR < 0.05. To minimize false positives, only SNPs identified by both methods were prioritized as high-confidence selection outliers.

Second, GEA analysis was performed to identify genomic regions specifically associated with environmental variation across the sampling locations. We analyzed five selected variables including elevation and four bioclimatic variables (BIO5, BIO6, BIO13 and BIO14), obtained from the WorldClim version 2.1 database at a 5 arc-minute resolution [76]. The relationship between environmental variation and genetic structure was explored via a multivariate Redundancy Analysis (RDA) [77, 78] using the *vegan* R package [67]. Outliers in the RDA were defined as loci residing ± 3 standard deviations (SD) from the mean loading of the significant axes [78].

To identify specific environment-associated outliers, we employed the latent factor mixed model (LFMM2) implemented in the LEA R package [79, 80], utilizing the non-imputed full dataset (MAF > 0.01, unpruned). To account for unobserved population structure, *K* = 6 latent factors were used, as determined by prior sparse non-negative matrix factorization (sNMF) analysis [81]. Statistical significance was assessed using Benjamini-Hochberg adjusted *p*-values (*p* < 0.05) [43] to identify significant environment-associated outliers. The genomic distribution and strength of these associations were visualized using Manhattan plots generated via the *qqman* R package [82]. Loci identified by both RDA and LFMM2 were considered putatively adaptive.

### Functional characterization of candidate loci

To understand the biological significance of the identified signals, functional annotation was conducted independently for the high-confidence selection outliers (the *F*_ST_ and pcadapt intersection) and the significant GEA-associated SNPs. All candidate SNPs were first mapped to the *R. leprosula* reference genome using BEDTools v2.30 [83] to identify associated genes.

These genes were functionally annotated using eggNOG-mapper v2 [84], which assigned Gene Ontology (GO) terms, Clusters of Orthologous Groups (COG) categories, protein domains and KEGG pathways based on the eggNOG orthology database. GO enrichment analysis was performed using the topGO v2.62 R package [85], to test for over-representation in Biological Process (BP), Molecular Function (MF) and Cellular Component (CC) ontologies. Statistical significance was determined using Fisher’s exact test with the ‘elim’ algorithm to account for the hierarchical structure of the GO terms. GO terms with *p* < 0.05 were considered significantly enriched. This independent evaluation allowed for a clear distinction between biological functions driving broad lineage divergence and those specifically involved in local adaptation to fine-scale environmental gradients.

## Results

### Genome-wide SNP discovery and distribution

Whole-genome resequencing of 194 *R. leprosula* individuals from 37 natural populations across Peninsular Malaysia, Sumatra and Borneo generated ~ 51.23 Gb of raw paired-end reads, with a mean coverage of ~ 16× per individual. Notably, more than 80.7% of the clean reads per individual were successfully mapped to the chromosome-scale *R. leprosula* reference genome (~ 440 Mb; Ng et al., unpublished data). Basic quality filtering of the 88.4 million raw variants yielded a primary pool of 1.1 million SNPs, which were partitioned into three functional datasets. A comprehensive set of 1,005,044 SNPs (MAF > 0.01) was finalized for selection-based analyses (outlier detection and GEA). For demographic and diversity assessments, two neutral subsets were established, comprising 1,004,005 (MAF > 0.01) and 803,934 (MAF > 0.05) SNPs. Finally, a highly refined subset of 230,803 independent, biallelic SNPs was generated for population structure analysis. This dataset represents a fully imputed genomic matrix characterized by a lack of redundant signals (*r*² < 0.1), ensuring the statistical independence of subsequent genetic differentiation analyses.

**Fig. 2:**
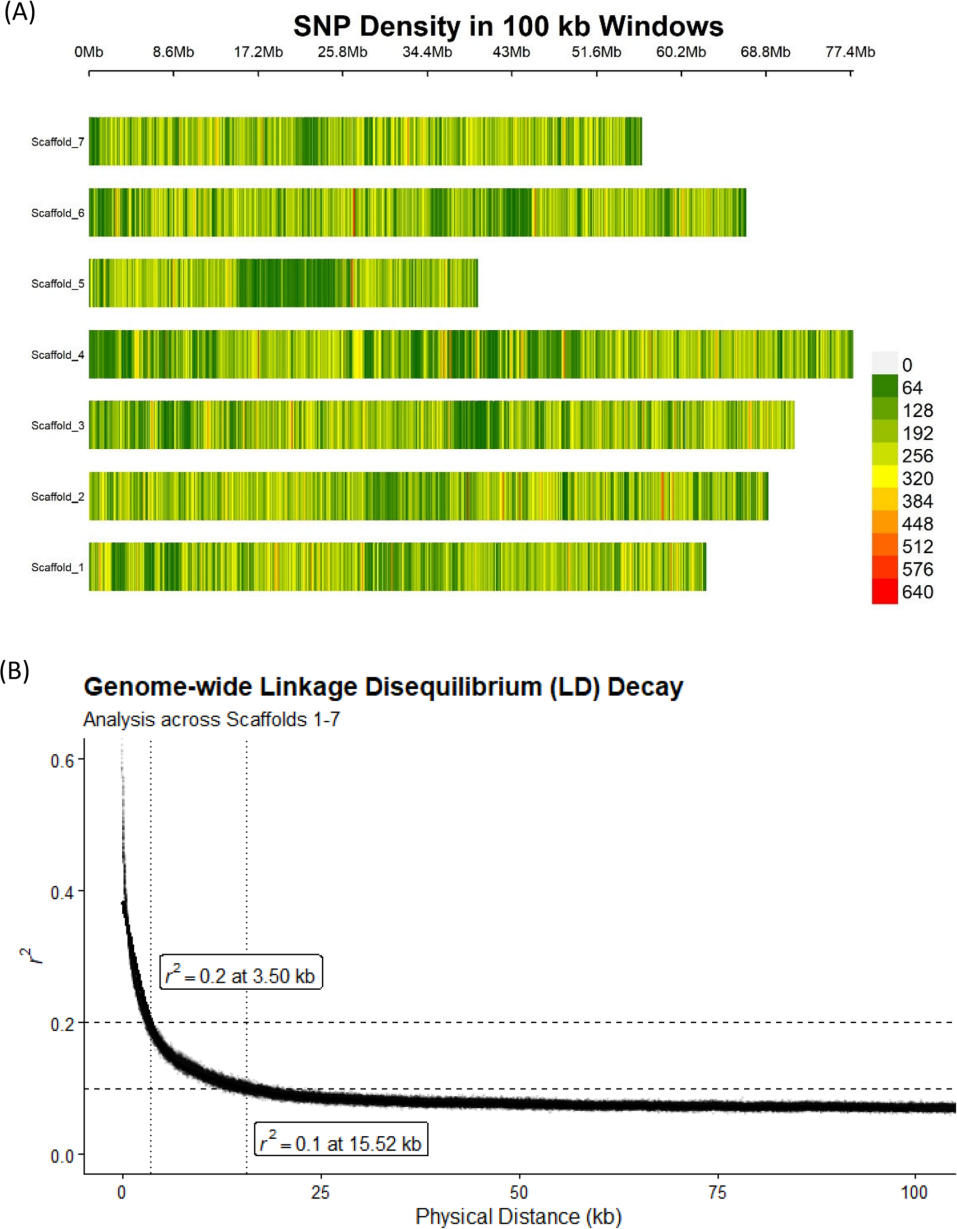
Genome-wide distribution of SNPs and linkage disequilibrium (LD) decay in *Rubroshorea leprosula*. **A** SNP density heatmap across the seven largest scaffolds of the *R. leprosula* reference genome, calculated in non-overlapping 100 kilobase (kb) windows. Warmer colours indicate higher SNP counts per window. **B** LD decay curve showing the decline of average pairwise *r*² with increasing physical distance between SNPs across Scaffolds 1–7. Thresholds are marked at *r*² = 0.2 and 0.1, indicating rapid LD decay within 3.50 kb and 15.52 kb, respectively

The genomic landscape across the seven primary scaffolds (39.5–80.0 Mb) exhibited a heterogeneous distribution of SNPs within 100 kb non-overlapping windows (Fig. 2A). Total SNP counts per scaffold ranged from 65,072 to 131,474, with localized densities fluctuating from < 64 to > 500 SNPs per 100 kb. Scaffolds 3, 4 and 6 harboured the highest frequency of high-density clusters. Notably, Scaffold 4 (~ 78 Mb) maintained a consistently elevated SNP density across its physical length, representing one of the most polymorphic sequences in the genome. In contrast, Scaffolds 2 and 7 displayed more moderate genetic variation, reflecting a non-random distribution of polymorphism across the *R. leprosula* genome.

### Linkage disequilibrium patterns and mapping resolution

Linkage disequilibrium (LD) analysis across Scaffolds 1–7 revealed a rapid decay of genetic correlation with increasing physical distance, indicating high genomic mapping resolution (Fig. 2B). Based on 804,797 high-quality SNPs, the average squared correlation coefficient (*r*^2^) declined to the critical threshold of 0.2 within 3.50 kb. The decay continued to a background level of *r*^2^ = 0.1 at 15.52 kb, eventually stabilizing at a negligible baseline of ~ 0.05 beyond 20 kb. This rapid decay profile is indicative of high historical recombination rates and a large effective population size (*N*_e_), consistent with an outcrossing mating system of *R. leprosula*. These empirical LD patterns established a robust framework for downstream analyses, specifically justifying the *r*^2^ < 0.1 pruning threshold required to ensure the statistical independence of markers for population structure and differentiation assessments.

### Patterns of genetic diversity and population neutrality

The summary of genetic diversity and neutrality tests across 37 populations is presented in Table 2. At the species-wide level, *R. leprosula* exhibited high genomic diversity, with a mean observed heterozygosity (*H*_O_) of 0.549 and nucleotide diversity (*π*) of 0.014. Across all populations, *H*_O_ consistently exceeded expected heterozygosity (*H*_E_), with the highest values observed in CS (0.676) and WK (0.669).

**Table 2:** Summary statistics of genetic diversity and neutrality tests across 37 populations and at the species-wide level for *Rubroshorea leprosula*. Observed heterozygosity (*H*_O_), expected heterozygosity (*H*_E_), nucleotide diversity (π), Watterson’s theta (*θ*_W_) and Tajima’s *D* were calculated at population level and species-wide. Values are presented as means ± standard deviations (SD)

Population	H_O_ (±) SD	H_E_ (±) SD	π (±) SD	θ_W_ (±) SD	Tajima’s D (±) SD
AHi	0.547 ± 0.009	0.396 ± 0.000	0.015 ± 0.011	0.018 ± 0.010	-0.686 ± 1.124
ERo	0.544 ± 0.008	0.394 ± 0.000	0.015 ± 0.011	0.018 ± 0.010	-0.664 ± 1.077
Lab	0.624 ± 0.011	0.469 ± 0.000	0.015 ± 0.012	0.022 ± 0.013	-1.839 ± 1.448
Mer	0.520 ± 0.019	0.374 ± 0.000	0.014 ± 0.010	0.017 ± 0.009	-0.479 ± 1.049
BSe	0.561 ± 0.008	0.401 ± 0.000	0.014 ± 0.011	0.018 ± 0.010	-0.655 ± 1.108
AKe	0.556 ± 0.021	0.391 ± 0.000	0.015 ± 0.011	0.016 ± 0.009	-0.228 ± 1.066
Ken	0.548 ± 0.003	0.396 ± 0.000	0.015 ± 0.011	0.018 ± 0.010	-0.610 ± 1.095
GAn	0.564 ± 0.052	0.393 ± 0.000	0.014 ± 0.010	0.017 ± 0.010	-0.537 ± 1.109
Ps	0.540 ± 0.004	0.392 ± 0.000	0.015 ± 0.011	0.017 ± 0.010	-0.492 ± 1.051
UGo	0.550 ± 0.006	0.396 ± 0.000	0.015 ± 0.011	0.018 ± 0.010	-0.606 ± 1.049
SLa	0.540 ± 0.001	0.384 ± 0.000	0.015 ± 0.011	0.016 ± 0.009	-0.093 ± 1.002
SMe	0.541 ± 0.018	0.383 ± 0.000	0.013 ± 0.010	0.017 ± 0.009	-0.531 ± 1.105
Kor	0.548 ± 0.005	0.397 ± 0.000	0.015 ± 0.011	0.018 ± 0.010	-0.593 ± 1.074
RBe	0.574 ± 0.008	0.421 ± 0.000	0.014 ± 0.011	0.020 ± 0.011	-1.185 ± 1.274
Tem	0.476 ± 0.143	0.362 ± 0.000	0.012 ± 0.010	0.018 ± 0.010	-1.277 ± 1.195
UKe	0.547 ± 0.011	0.387 ± 0.000	0.015 ± 0.011	0.016 ± 0.009	-0.139 ± 1.033
GIn	0.500 ± 0.121	0.377 ± 0.000	0.013 ± 0.010	0.019 ± 0.011	-1.399 ± 1.298
SBa	0.558 ± 0.013	0.400 ± 0.000	0.014 ± 0.011	0.018 ± 0.010	-0.795 ± 1.173
BEn	0.539 ± 0.071	0.383 ± 0.000	0.015 ± 0.011	0.017 ± 0.009	-0.174 ± 0.997
SBe	0.553 ± 0.002	0.397 ± 0.000	0.015 ± 0.011	0.018 ± 0.010	-0.631 ± 1.109
CTo	0.563 ± 0.013	0.401 ± 0.000	0.014 ± 0.011	0.018 ± 0.010	-0.756 ± 1.164
USa	0.502 ± 0.104	0.368 ± 0.000	0.013 ± 0.010	0.017 ± 0.009	-0.503 ± 1.055
BKe	0.556 ± 0.010	0.400 ± 0.000	0.014 ± 0.011	0.018 ± 0.010	-0.731 ± 1.160
HTe	0.515 ± 0.086	0.371 ± 0.000	0.013 ± 0.010	0.017 ± 0.010	-0.869 ± 1.124
Bes	0.550 ± 0.012	0.396 ± 0.000	0.014 ± 0.011	0.018 ± 0.010	-0.679 ± 1.109
Jen	0.537 ± 0.008	0.391 ± 0.000	0.015 ± 0.011	0.018 ± 0.010	-0.594 ± 1.060
TNe	0.616 ± 0.012	0.466 ± 0.000	0.014 ± 0.012	0.023 ± 0.013	-2.268 ± 1.594
Len	0.537 ± 0.007	0.382 ± 0.000	0.015 ± 0.011	0.016 ± 0.009	-0.216 ± 1.022
CS	0.676 ± 0.034	0.442 ± 0.000	0.012 ± 0.010	0.017 ± 0.010	-0.972 ± 1.283
NS	0.532 ± 0.024	0.379 ± 0.000	0.014 ± 0.010	0.017 ± 0.009	-0.421 ± 1.051
WK	0.669 ± 0.036	0.445 ± 0.000	0.012 ± 0.010	0.017 ± 0.010	-1.181 ± 1.235
LK	0.467 ± 0.085	0.345 ± 0.000	0.011 ± 0.009	0.018 ± 0.010	-1.512 ± 1.152
Dan	0.568 ± 0.007	0.401 ± 0.000	0.011 ± 0.010	0.018 ± 0.010	-1.533 ± 1.220
Imb	0.592 ± 0.059	0.413 ± 0.000	0.012 ± 0.010	0.018 ± 0.010	-1.448 ± 1.289
Mal	0.565 ± 0.005	0.400 ± 0.000	0.012 ± 0.010	0.018 ± 0.010	-1.476 ± 1.226
GGa	0.490 ± 0.207	0.375 ± 0.000	0.011 ± 0.009	0.019 ± 0.011	-1.672 ± 1.225
KMa	0.583 ± 0.009	0.409 ± 0.000	0.012 ± 0.010	0.018 ± 0.010	-1.315 ± 1.225
Species-wide	0.549 ± 0.046	0.395 ± 0.029	0.014 ± 0.001	0.018 ± 0.001	-0.858 ± 0.522

Variation in intra-population heterozygosity differed among populations. In particular, GGa (SD = 0.207), Tem (SD = 0.143) and GIn (SD = 0.121) showed relatively high standard deviations in *H*o, indicating significant genotypic heterogeneity within these populations. A pronounced spatial pattern in genomic diversity was observed across the study region. Populations from Peninsular Malaysia (e.g., Lab: *H*_O_ = 0.624, TNe: *H*_O_ = 0.616, RBe: *H*_O_ = 0.574) generally exhibited higher heterozygosity than several populations from Borneo (e.g., GGa: *H*_O_ = 0.490, LK: *H*_O_ = 0.467).

Genome-wide average nucleotide diversity (π), Watterson’s theta (*θ*_W_), and Tajima’s *D* values were estimated as 0.014, 0.018 and − 0.858, respectively (Table 2), which was comparable to a previous study that used fewer nuclear loci [26].

### Population structure and phylogenetic relationships

**Fig. 3:**
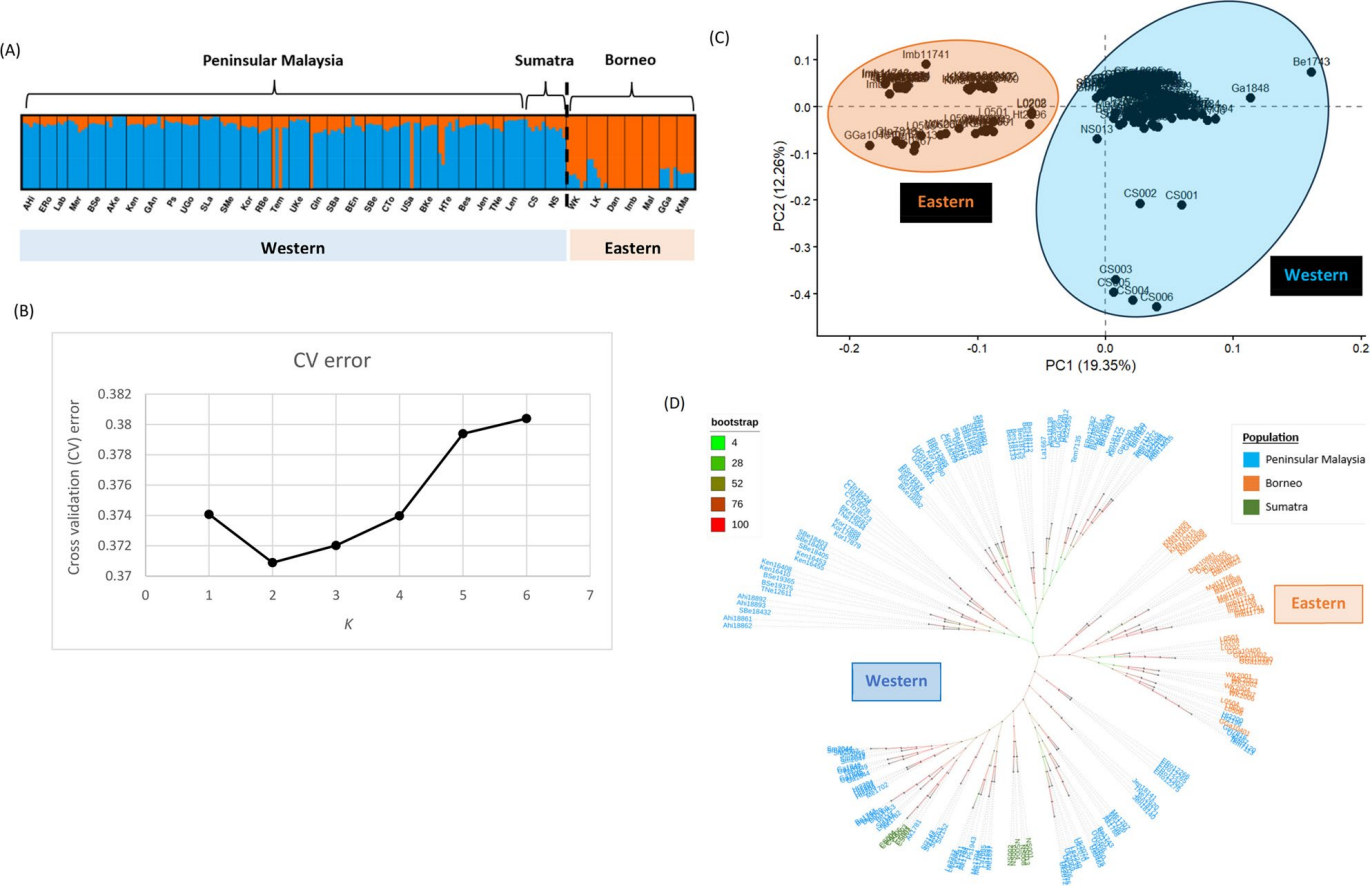
Genetic structure and evolutionary relationships among *Rubroshorea leprosula* individuals based on the pruned neutral SNP dataset. **A** Global ADMIXTURE bar plot at *K* = 2 showing distinct ancestries for Cluster A (blue) and Cluster B (orange). **B** Cross-validation (CV) error plot showing the lowest error at *K* = 2, indicating the optimal number of ancestral populations. **C** Principal Component Analysis (PCA) plot with the first two components (31.61% variance) enclosed by 95% confidence ellipses. PC1 separates the Eastern and Western lineages, with the six admixed individuals clustering within the Eastern ellipse. **D** Maximum-likelihood (ML) phylogenetic tree supporting the deep East-West divergence. The tree exhibits high bootstrap support for the primary clades and reveals the monophyletic grouping of the six admixed individuals within the Eastern clade

Genetic structure analysis using the independent, neutral SNP dataset identified *K* = 2 as the optimal number of ancestral clusters (Fig. 3A; Supplementary Fig. S1A), supported by the minimum cross-validation (CV) error (Fig. 3B). This primary split defined a deep biogeographic divergence across the Sunda Shelf, partitioning populations into a Western lineage (Cluster A; Peninsular Malaysia and Sumatra) and an Eastern lineage (Cluster B; seven Bornean populations). A nested ADMIXTURE approach further resolved the hierarchical architecture masked by this primary divergence. At *K* = 3, the Sumatran (CS) and Western Bornean (WK) populations emerged as distinct genetic components (Supplementary Fig. S1A). Subset analyses confirmed this regional sub-structure, identifying CS as a unique lineage separate from the relatively homogeneous Peninsular/Sumatra group and highlighting significant internal heterogeneity within Borneo, localized predominantly in the WK population (Supplementary Fig. S1B).

These findings were further corroborated by Principal Component Analysis (PCA), with the first two components accounting for 31.61% of the total genetic variance (Fig. 3C). While PC1 (19.35%) delineated the Eastern and Western lineages, PC2 (12.26%) further differentiated the Sumatran (CS) individuals, mirroring the nested ADMIXTURE results. Despite the strong geographic clustering, a distinct transition zone was identified in northern Peninsular Malaysia. Six individuals (HTe2196, HTe2200, USa0467, Tem7113, Tem7120 and GIn7816) exhibited mixed ancestry and clustered within the Bornean ellipse, indicating a genome-wide affinity to Eastern populations despite their Western geographic origin.

The ML phylogenetic tree (Fig. 3D) provided robust validation of these structural divisions, exhibiting a deep primary split between Western and Eastern clades with high bootstrap support. Within the Western clade, the CS population formed a distinct monophyletic group, while the six admixed Peninsular individuals grouped monophyletically within the Bornean clade. The overall topology was characterized by a star-like radiation with long terminal branches relative to the short internal nodes.

### Genetic differentiation, AMOVA and isolation by distance

Pairwise *F*_ST_ analysis of the 37 populations of *R. leprosula* showed values ranging from 0.000 to 0.075, with an overall mean of 0.032 (Supplementary Fig. S2). Notably, more than half of the comparisons showed no measurable genetic divergence (*F*_ST_ = 0.000) after adjusting negative estimates to zero. Despite this general similarity, the highest divergence (*F*_ST_ = 0.075) was consistently found between the most geographically distant populations. Additionally, a distinct cluster of Bornean populations showed moderate separation (*F*_ST_ > 0.040) when compared to the populations from Peninsular Malaysia.

**Fig. 4:**
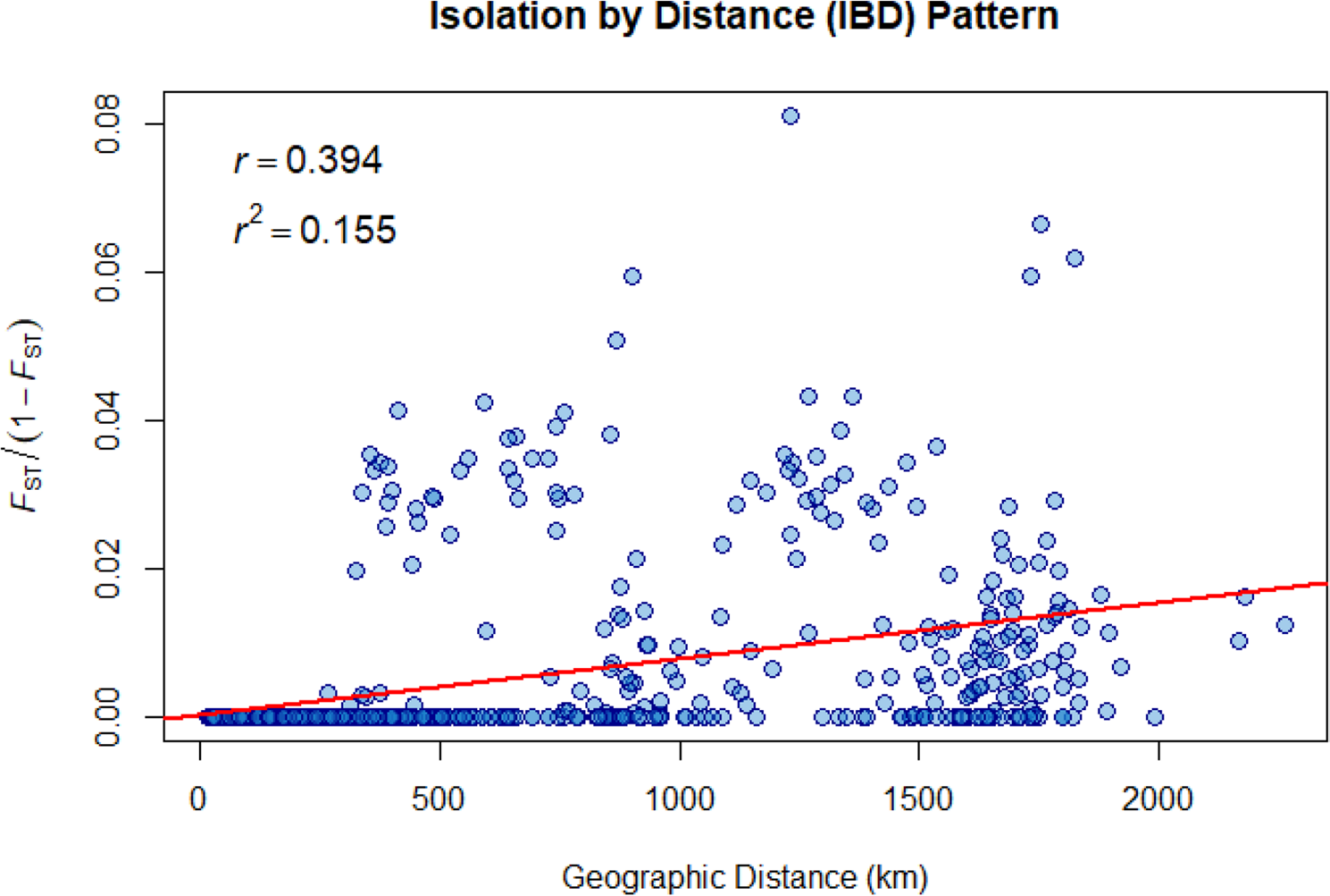
Isolation by distance (IBD) among 37 populations of *R. leprosula*. The scatter plot illustrates the correlation between linearized genetic distance and geographic distance (km). The Mantel test indicates a significant positive correlation (*r* = 0.394, *p* < 0.001), with geographic distance explaining 15.5% (*r*² = 0.155) of the total observed genetic variation

The AMOVA results confirmed that most of the genetic variation is found within populations (96.58%), with only 3.42% partitioned among populations (Table 3). This global fixation index was statistically significant (ΦST = 0.034, *p* < 0.001). The Mantel test confirmed a significant positive correlation between linearized genetic distance and geographic distance (*r* = 0.394, *p* < 0.001) (Fig. 4). Geographic distance explained approximately 15.5% (*r*^2^ = 0.155) of the genetic differentiation observed.

**Table 3:** AMOVA results for *Rubroshorea leprosula* based on genome-wide SNP data. The AMOVA partitions genetic variation among and within populations

Source of Variation	Df	SS	Variance Component (σ²)	Variation (%)	Phi Statistic	*p*-value
Among populations	36	1,145,382	1,157.95	3.42	ΦST = 0.034	0.001
Within populations	351	11,966,903	32,719.26	96.58	–	–
Total	387	13,112,284	33,877.21	100.00	–	–

All levels showed significant structure based on 999 permutations

*Df*  Degrees of freedom, *SS*  Sum of squares

### Historical demographic inference

**Fig. 5:**
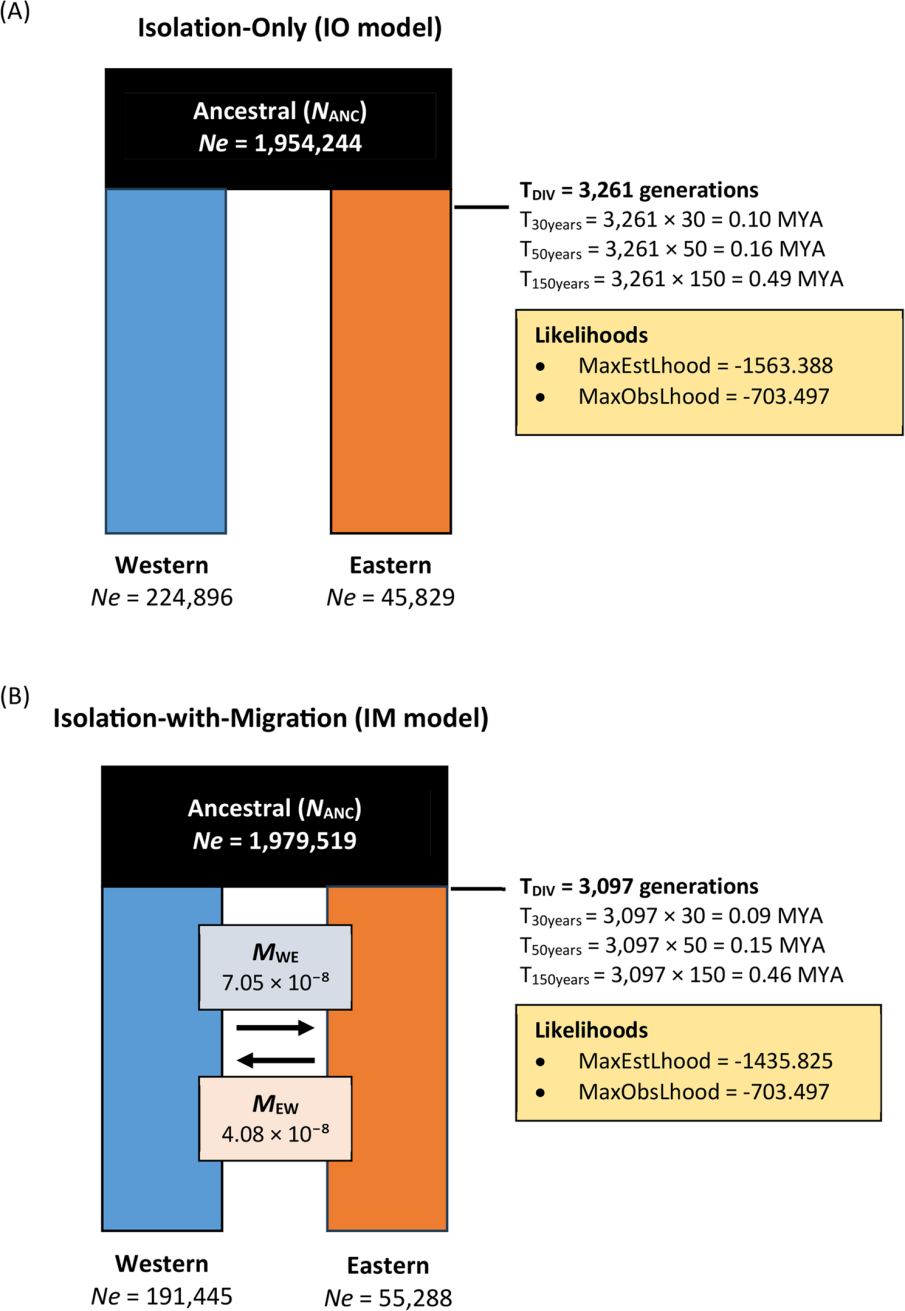
Schematic demographic divergence models between Western and Eastern clusters of *Rubroshorea leprosula*. **A** Isolation-only (IO) model showing divergence without post-divergence gene flow. **B** Isolation-with-migration (IM) model indicating asymmetric post-divergence gene flow. Effective population size (*Ne*) and divergence time (T_DIV_, in generations) are shown for each population and ancestral lineage

Demographic modelling identified the Isolation-with-Migration (IM) model as the best-fitting representation of the history of *R. leprosula* (Fig. 5). This model achieved a higher maximum composite likelihood compared to the Isolation-Only (IO) model (-1435.83 vs. -1563.39) and a substantially lower AIC value (2885.65 vs. 3136.78, ΔAIC = 251.13).

Under the preferred IM model, the current effective population sizes (*N*_e_) were estimated at 191,445 for the Western cluster and 55,288 for the Eastern cluster. The ancestral effective population size (*N*_*ANC*_) was significantly larger (1,979,519), reflecting a substantial population contraction after divergence, with an ancestral-to-descendant size ratio (*RESIZE*_WE_) of 10.34. The estimated mutation rate was 8.75 × 10⁻⁹ substitutions per site per generation. Divergence time was estimated at 3,097 generations, corresponding to 0.46 − 0.09 MYA (assuming a generation time of 30–150 years). Additionally, the IM model recovered asymmetric post-divergence gene flow, with a higher migration rate from the Western to the Eastern cluster (7.05 × 10⁻⁸) than the reverse (4.08 × 10⁻⁸).

### Genomic scans for divergent selection and functional analysis

**Fig. 6:**
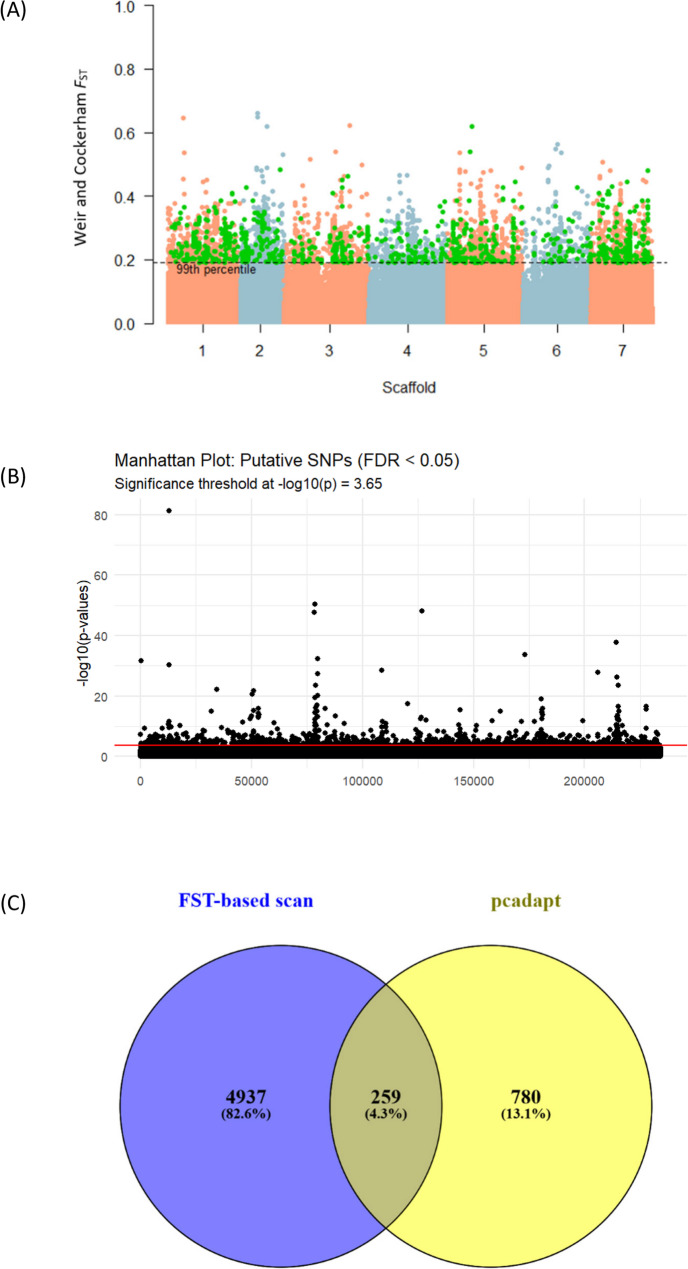
Genome-wide identification of consensus selection outliers. **A** Manhattan plot of pairwise *F*_ST_ between Western and Eastern clusters, with a black dashed line denotes the 99^th^ percentile threshold. **B** Manhattan plot of pcadapt scores with a red significance threshold of FDR < 0.05. **C** Venn diagram showing the intersection of both methods, resulting in 259 high-confidence consensus SNPs prioritized for downstream local adaptation analysis

A genome-wide *F*_ST_ scan based on 514,040 SNPs across Scaffolds 1–7 identified 5,196 loci (top 1%) that were highly differentiated between the Western and Eastern clusters (Fig. 6A). Simultaneously, the principal component-based approach in pcadapt identified 1,039 putative outliers driving structural genomic variation across the same scaffolds (Fig. 6B). To minimize false positives, we prioritized the intersection of these two independent methods, yielding 259 high-confidence selection outliers (Fig. 6C). This refined subset represents genomic regions where strong lineage divergence aligns with statistically significant structural variation.

Functional annotation assigned the genes associated with these high-confidence outliers to 18 major Clusters of Orthologous Groups (COG) categories, primarily involving signal transduction, transcription and post-translational modifications (Supplementary Figure S3A). Gene Ontology (GO) analysis yielded 13,279 terms, with dominant Biological Process (BP), Cellular Component (CC) and Molecular Functions (MF) categories associated with cellular metabolism, intracellular structure and catalytic or binding activity (Supplementary Figure S3B). Further enrichment analysis revealed that the top 35 GO terms were specifically linked to abiotic stress responses, including temperature, osmotic pressure and water deprivation (Supplementary Figure S3C). KEGG analysis linked these adaptive loci to 116 pathways, with significant enrichment in energy metabolism (TCA cycle, glycolysis and photosynthesis) and intracellular signalling (MAPK and plant hormone signal transduction) (Supplementary Figure S3D).

### Genomic signatures of environmental adaptation and functional analysis

Environmental analysis revealed a significant climatic mosaic across the range of *R. leprosula*, characterized by a strong thermal-altitudinal gradient and spatially heterogeneous precipitation patterns (Supplementary Table S1; Supplementary Fig. S4).

**Fig. 7:**
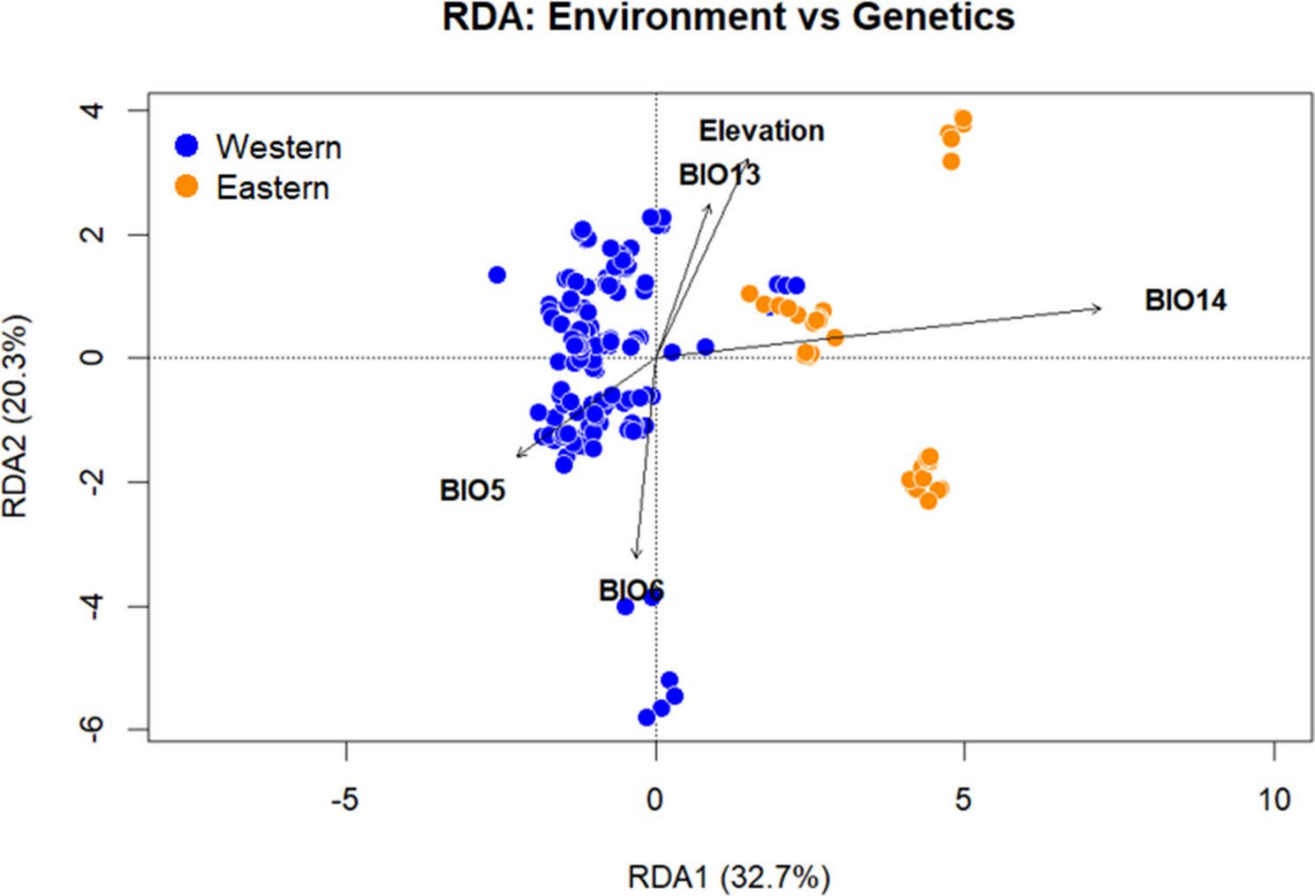
Redundancy Analysis (RDA) biplot of genomic variation in *R. leprosula* constrained by environmental variables. Each point represents an individual sample, coloured by genomic group: Western cluster (blue) and Eastern cluster (dark orange). Black vectors indicate the direction and strength of environmental variables: BIO5 (Max Temperature of Warmest Month), BIO6 (Min Temperature of Coldest Month), BIO13 (Precipitation of Wettest Month), BIO14 (Precipitation of Driest Month) and Elevation. Values in parentheses represent the percentage of constrained variance explained by the first two axes (RDA1 and RDA2)

The RDA biplot revealed a clear genomic separation between the Western and Eastern clusters along the first two axes (RDA1 = 32.7% and RDA2 = 20.3%; Fig. 7). The Western cluster was primarily associated with higher temperatures (represented by BIO5 and BIO6), while the Eastern cluster showed a stronger association with variables related to elevation and precipitation seasonality (BIO13). Notably, BIO14 (precipitation of the driest month) emerged as a distinct vector, suggesting that water availability shapes genetic differences of *R. leprosula* independently, without being tied to the usual changes in temperature or elevation.

**Fig. 8:**
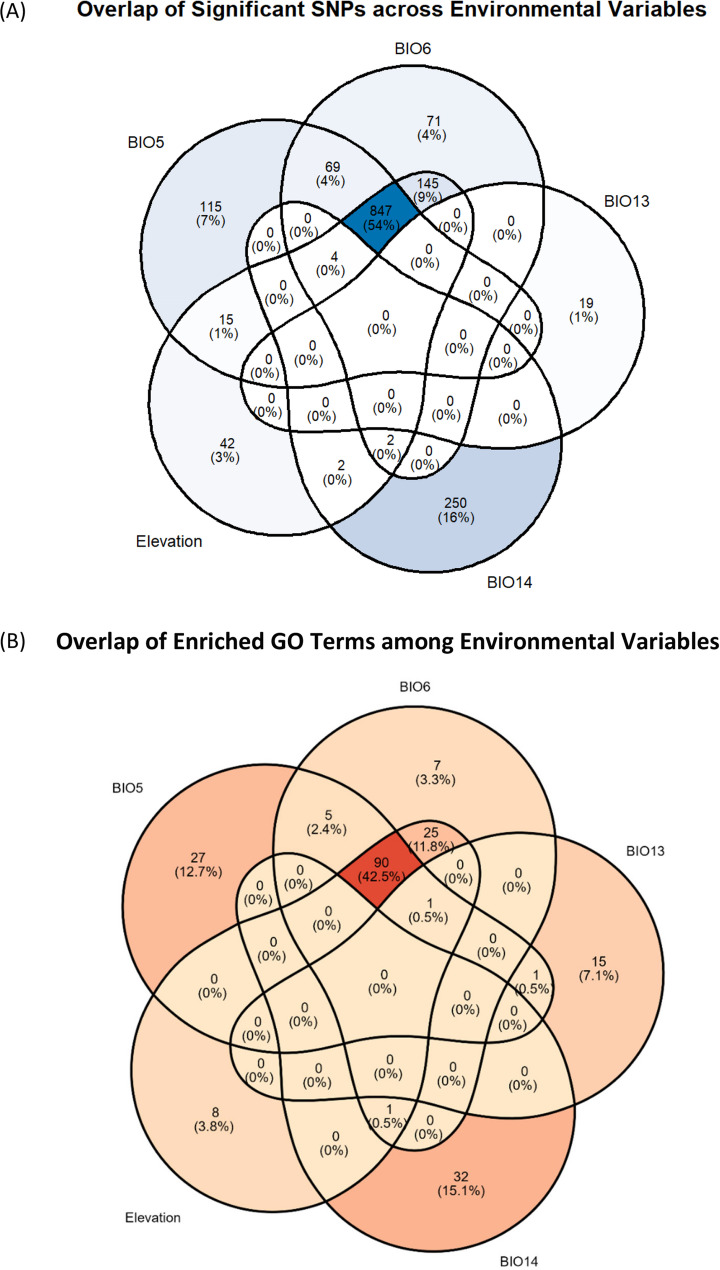
Venn diagrams showing overlaps among five environmental variables (BIO5, BIO6, BIO13, BIO14 and elevation) in *Rubroshorea leprosula*. **A** Overlap of significant SNPs identified from genome-environment association (GEA) analyses. **B** Overlap of enriched Gene Ontology (GO) terms associated with genes linked to these environmental variables

Genome-environment association (GEA) analyses via LFMM2 identified a dominant genomic signature associated with the temperature-elevation gradient. The strongest associations were observed for BIO5 (1,050 SNPs), BIO6 (1,138 SNPs) and elevation (1,057 SNPs), with a significant triple-overlap of 847 SNPs (54%) identifying a unified selective force (Fig. 8A). In contrast, precipitation variables exhibited substantially fewer and more localized associations (BIO13: 19 SNPs; BIO14: 258 SNPs) with minimal overlap. Individual Manhattan plots for each bioclimatic variable are provided in Supplementary Fig. S5.

**Fig. 9:**
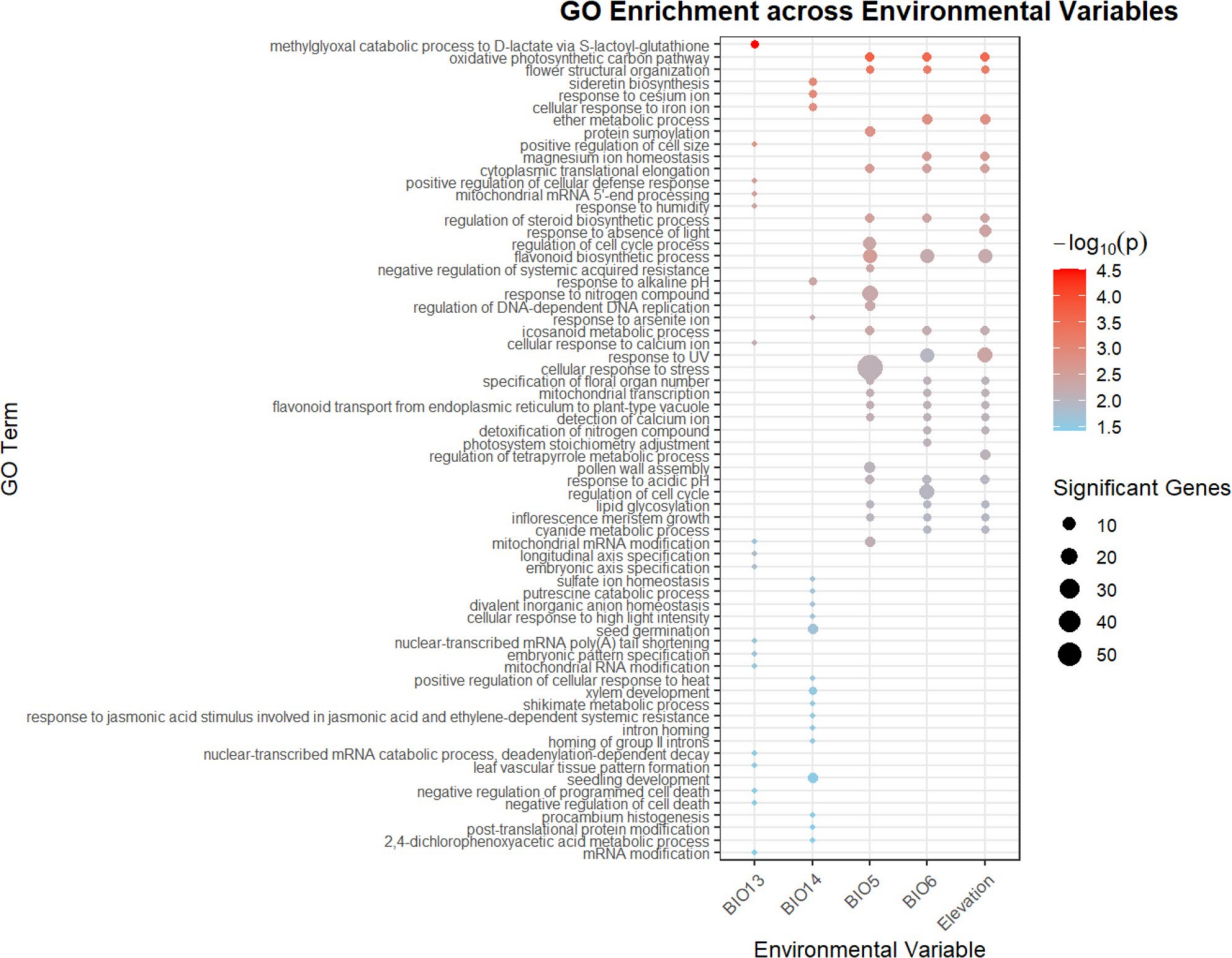
Top 20 enriched GO terms (*p* < 0.05) for each environmental variable (BIO5, BIO6, BIO13, BIO14 and elevation). Bubble size represents the number of significant genes per GO term, while colour intensity indicates enrichment significance (-log_10_ *p*)

Functional enrichment analyses of the congruent SNP set (defined as the intersection of outliers from both RDA and LFMM2) supported these genomic patterns. These high-confidence adaptive-outlier SNPs associated with temperature and elevation shared 90 enriched GO terms (42.5%), highlighting a concerted adaptive response involving photosynthesis, oxidative metabolism and photoprotection (e.g., oxidative photosynthetic carbon pathway and UV stress response; Figs. 8B and 9). Conversely, precipitation variables yielded highly specific and non-overlapping functional profiles. BIO13 was uniquely enriched for developmental regulation and immune-related responses, while BIO14 was linked to ion homeostasis, seed germination and drought-related signalling (Fig. 9).

## Discussion

### Genomic diversity and population structure

*Rubroshorea leprosula* exhibits high genomic diversity, consistent with expectations for a widespread, predominantly outcrossing tropical tree species [21]. The consistent excess of observed (*H*_O_) over expected heterozygosity (*H*_E_) across hierarchical levels, indicating a pattern of heterozygote excess. This pattern may reflect biological processes likely explained by selection against inbred individuals during the long-life cycle and mechanisms that promote outcrossing, such as self-incompatibility systems, but could also arise from admixture among differentiated lineages [21, 86–88]. In addition, the observed genetic diversity likely represents a legacy of historical forest connectivity, as the long lifespan of these trees creates a time lag that can hide the genetic impact of recent anthropogenic pressures such as habitat fragmentation.

Genome-wide nucleotide diversity (*π* = 0.011 to 0.015) indicates substantial standing genetic variation across populations, consistent with a large long term effective population size and wide ecological distribution. Such levels of diversity are expected to confer considerable adaptive potential under environmental change [89, 90]. However, in a broader comparative context, these values are moderate relative to some dipterocarps in marginal Asian rainforests; for example, species from *Vatica* genera exhibit substantially higher nucleotide diversity (*π*) from 0.039 to 0.094 for *V. guangxiensis* and *V. mangachapoi*, respectively [10]. The relatively narrow range of *π* across the 37 populations suggests a broadly even distribution of genetic variation, emphasizing the importance of maintaining population connectivity to preserve evolutionary potential [89, 90], particularly in dipterocarps where fragmentation can rapidly erode genomic diversity [10].

Population structure analyses based on genome-wide SNPs revealed two major genetic clusters: Western (Peninsular Malaysia and Sumatra) and Eastern (Borneo), reflecting the well-established west-east biogeographic partition of Sundaland, shaped by repeated cycles of connection and isolation driven by Pleistocene sea-level fluctuations [19, 26, 91]. Although land bridges periodically connected these regions, palaeoecological evidence suggests that large portions of the exposed shelf were dominated by non-forest habitats, which would have limited dispersal for forest-dependent species [16, 19, 91–93]. Subsequent interglacial sea-level rise re-established marine barriers, reinforcing divergence and contributing to the distinct genomic signature of Bornean populations.

This historical isolation is further reflected in a pronounced west-east gradient of genomic diversity. Populations from Peninsular Malaysia (e.g., Lab, TNe and RBe) and Sumatra (CS) exhibit higher heterozygosity than those from Borneo (e.g., GGa and LK), likely reflecting historically larger effective population sizes (*N*_e_) and greater connectivity among western landmasses during glacial maxima. While the Western region functioned as a genetic reservoir, the lower diversity in Borneo is consistent with prolonged isolation and more localized demographic trajectories [5, 24, 94]. Despite this clear regional structure, evidence of localized admixture indicates that populations boundaries are not absolute. Individuals in northern Peninsular Malaysia showing partial Bornean ancestry may reflect historical secondary contact following range expansions or the retention of ancestral polymorphisms.

### Regional differentiation and historical connectivity

The predominance of genetic variation within populations (96.58%) is consistent with expectations of long-lived, outcrossing tropical trees species like *R. leprosula* [21, 86, 87, 95], reflecting historical gene flow and large effective population sizes that maintain substantial standing diversity [19, 94, 96]. The significant isolation-by-distance (IBD) signal further support geographically restricted gene flow, however, the modest variance explained (*r*^2^ = 0.155) and the occurrences of near low *F*_ST_ values among populations suggest that contemporary distance alone cannot account for the observed patterns. Instead, the genetic structure likely reflects an interplay between ongoing gene flow or historical connectivity across the Sunda Shelf, where repeated exposure of land bridges during Pleistocene low sea levels facilitated gene flow between now-disjunct regions such as Peninsular Malaysia, Sumatra and Borneo. Comparable patterns of weak structure coupled with IBD have been reported in other dipterocarps, including *Dryobalanops aromatica* [97], *R. curtisii* [98] and *R. parvifolia* [94], reinforcing the generality of these processes across the region. These findings highlight that both spatially restricted gene flow and historical landscape dynamics have shaped the contemporary genetic landscape of *R. leprosula*, underscoring the importance of maintaining landscape connectivity to preserve gene flow and support long-term evolutionary potential.

Demographic inference provides the historical context into the temporal processes underlying this structure. The large ancestral effective population size (*N*_ANC_ ~1.95–1.98 million), followed by a pronounced 10-fold reduction in the descendant populations (*RESIZE*_WE_ ≈ 8.7–10.3×), indicates a substantial contraction after the divergence, estimated to have occurred during the Mid- to Late-Pleistocene (0.46 − 0.09 MYA). This period was characterized by repeated glacial-interglacial cycles and pronounced sea levels fluctuations across the Sunda Shelf [15, 19, 99], which would have alternately facilitated connectivity and enforced isolation. The estimated timing is consistent with life history traits of dipterocarps, which are long-lived with extended generation times; while Ashton [100] proposed a 60-year minimum generation time for *Shorea*, others report first-flowering at 20–30 years [101]. Signals of demographic recovery are supported by consistently negative Tajima’s *D* values across populations, indicative of recent population expansion, likely originating from stable humid refugia in northern Peninsular Malaysia and Borneo [19, 93, 102, 103]. However, spatial variation in neutrality statistics suggests that expansion dynamics were heterogeneous, likely reflecting localized histories of persistence, isolation and recolonization. These neutrality statistics should therefore be interpreted as complementary to, rather than independent evidence of, the broader demographic patterns.

Model-based inference further clarifies the role of gene flow in shaping population history. The strong support for an IM model (ΔAIC = 251.13) indicates that divergence occurred with subsequent secondary contact rather than complete isolation. This interpretation is corroborated by PCA and ADMIXTURE analyses, which show clear signals of post-divergence admixture. Notably, gene flow appears asymmetric, with higher migration rates from the Western populations (Peninsular Malaysia/Sumatra) to the Eastern populations (Borneo). This suggests that Western populations may have acted as demographic sources during periods of low sea level, when exposed land bridges across the Sunda Shelf enabled episodic connectivity [15, 91, 94, 99]. Such connectivity highlights the profound influence of Pleistocene sea-level cycles in structuring population connectivity and driving the spatial genetic patterns observed in Southeast Asian dipterocarps.

### Signatures of local adaptation and genomic divergence

The application of the top 1% *F*_ST_ threshold and pcadapt effectively identified candidate loci in the extreme tail of genomic differentiation, which are most likely influenced by natural selection rather than genetic drift [74, 104, 105]. Functional enrichment of these outlier-associated genes suggests that adaptive divergence in *R. leprosula* is primarily driven by mechanisms of environmental sensing and physiological homeostasis. Specifically, the enrichment of COG categories related to signal transduction and post-translational modifications including protein kinases and molecular chaperones reflects the species’ capacity to modulate cellular responses under environmental stress [106, 107]. This is further supported by GO enrichment analysis, which revealed an overrepresentation of genes mediating tolerance to water deprivation, temperature extremes and light variation, highlighting the diverse physiological challenges *R. leprosula* faces across its altitudinal and microclimatic range.

Crucially, the enrichment of hormone-related terms such as response to auxin, abscisic acid and jasmonic acid underscores the pivotal role of hormonal signalling in regulating these environmental stress responses and maintaining developmental plasticity across heterogeneous landscapes.

These findings are further complemented by KEGG pathway analysis, where the representation of ubiquitin-mediated proteolysis, fatty acid biosynthesis and phenylpropanoid pathways indicates robust mechanisms for protein turnover, membrane remodelling and oxidative stress mitigation. Additionally, the enrichment of pathways associated with regulation of the actin cytoskeleton and endocytosis suggests that cytoskeletal and membrane dynamics are key components of cellular adaptation to fluctuating environments. The significant representation of major signalling pathways, including MAPK, AMPK and mTOR, highlights their integrative roles in mediating cellular responses to stress, energy balance and growth regulation [107, 108]. Finally, the presence of circadian rhythm and hormone signalling pathways underscores that adaptation in *R. leprosula* involves not only physiological tolerance but also phenological regulation, enabling the species to synchronize growth and reproductive cycles with environmental cues such as light intensity and rainfall periodicity [109, 110].

### Environmental gradients shaping genomic structure and adaptation

Redundancy Analysis (RDA) indicates that elevation is a primary driver of genomic variation across the study area, with temperature variables (BIO5 and BIO6) aligning strongly yet inversely along the elevation gradient. In contrast, precipitation variables (BIO13 and BIO14) appear largely independent of elevation, suggesting that thermal and hydrological regimes act as distinct ecological pressures. The high proportion of variance explained by the first two RDA axes (53% total) supports the significance of these climatic factors, implying that populations experience multiple, simultaneous selective pressures. This mirrors patterns in other tropical systems, where temperature serves as a key driver of ecological specialization [111, 112].

The RDA biplot confirms that these environmental gradients structure genomic variation differently across the species’ range. Western populations (Peninsular Malaysia and Sumatra) were primarily associated with warmer, thermally stable lowland conditions [10, 111]. Conversely, Bornean populations exhibit a stronger association with higher elevations and wet-season precipitation (BIO13), reflecting adaptation to cooler upland regions where orographic rainfall is more prominent [113]. These spatial patterns suggest that while temperature is a dominant environmental correlate in the Western cluster, hydrological regimes play a more significant role in shaping the genomic divergence of Bornean populations.

Consistent with these environmental patterns, genome-environment association (GEA) analyses using LFMM2 identified a higher number of SNPs significantly associated with temperature and elevation compared to precipitation. Notably, the strong overlap of 847 SNPs associated with BIO5, BIO6 and elevation suggests a polygenic response to the temperature-altitude gradient, rather than adaptation through single major-effect loci. Such polygenic adaptation, characterized by subtle allele frequency changes across many loci, is common in long-lived tropical trees with large population sizes and high gene flow, as seen in *Pinus pinaster* [114] and high-altitude conifers such as coast redwood (*Sequoia sempervirens*) and giant sequoia (*Sequoiadendron giganteum*) [115]. For *R. leprosula*, this polygenic architecture likely facilitates a flexible adaptive response to the heterogeneous and topographically complex landscapes of Sundaland.

Functional enrichment analysis reinforces the GEA findings, revealing that SNPs associated with temperature and elevation are enriched for GO terms related to energy metabolism, oxidative stress response and photoprotection. Specifically, enrichment in the oxidative photosynthetic carbon pathway, flavonoid biosynthesis and UV response suggests that *R. leprosula* adapts to altitudinal gradients by optimizing photosynthetic efficiency and antioxidant defense. These mechanisms are essential for maintaining homeostasis under the high-intensity light and thermal fluctuations characteristic of upland environments.

Additionally, the enrichment of steroid biosynthetic and signalling pathways indicates that these temperature-driven physiological shifts are mediated by complex hormonal regulation. In contrast, adaptation to precipitation variables appears driven by gene regulatory plasticity and osmotic balance. Enrichment in GO terms related to mRNA modification, seed germination and ion homeostasis suggests that *R. leprosula* manages rainfall variability and seasonal drought through precise cellular and developmental regulation. Ng et al. [5] identified drought-responsive gene families in the species, and broader studies across the Dipterocarpoideae showing positive selection in antioxidation and metabolic pathways [6].

These consistent findings indicate that *R. leprosula* and other dipterocarps exhibit polygenic adaptation to environmental variation, driven by the modulation of stress-response and metabolic pathways. The alignment of genomic structure with environmental gradients further suggests that both historical population isolation and ecological adaptation have jointly shaped the genomic landscape of this tropical tree.

### Implications for conservation and adaptive potential

The identified GEAs provide a critical baseline for understanding the historical adaptive capacity of *R. leprosula*. These findings indicate that the species has survived environmental heterogeneity by developing specialized climatic niches (specific zones of temperature and rainfall). However, the genomic differentiation between clusters suggests that the genetic resources available for responding to environmental change differ by region. Populations occupying narrow climatic ranges particularly those adapted to the cooler, high-elevation habitats of Borneo may be vulnerable if future conditions shift rapidly beyond the thermal limits that have historically structured their genomic variation.

From a management perspective, these results underscore the importance of preserving genetic representativeness. Identifying populations with distinct adaptive variants allows forest managers to prioritize evolutionary hotspots that have maintained high diversity through Pleistocene fluctuations. Maintaining landscape connectivity remains essential to ensure that the historical adaptive potential of *R. leprosula* is not lost to fragmentation, providing a scientific basis for sustaining the species’ long-term evolutionary legacy across Southeast Asia.

## Conclusions

This study provides a genome-wide assessment of population structure, historical demography and environmental associations in *R. leprosula* across its natural range. High-density SNP data revealed a pronounced genetic divergence between east and west, shaped by isolation and demographic histories. The integration of genomic outlier detection and environmental association analyses identified two distinct yet complementary signatures of selection within the *R. leprosula* genome. The first set identifies genomic regions driven by divergent selection between regional lineages, while the second set highlights a polygenic adaptation to specific temperature, elevation, and precipitation gradients. Functional enrichment of these candidates highlights a shared focus on pathways related to photosynthesis, oxidative stress and hormonal signalling. This indicates that *R. leprosula* survives environmental heterogeneity by modulating its physiological response to light and thermal stress. These findings define the specific climatic niches and genetic resources that sustain regional lineages. Ultimately, this research provides a robust framework for conservation, emphasizing that preserving the genetic representativeness of these divergent clusters is essential for maintaining the species’ long-term adaptive potential across Southeast Asia.

## Supplementary Information

Supplementary Fig. S1. Supplementary Fig. S2. Supplementary Fig. S3. Supplementary Table S1. Supplementary Fig. S4. Supplementary Fig. S5.

## Data Availability

The reference genome assembly and associated raw reads are available in the DDBJ/EMBL/GenBank databases under BioProject PRJDB8161. The raw whole-genome resequencing reads generated for all individuals in this study have been deposited to the NCBI Sequence Read Archive (SRA) under BioProject PRJNA1223480.

## Abbreviations

*R.**leprosula*: *Rubroshorea leprosula*
SNP: Single nucleotide polymorphism
GEA: Genome-environment association
IBD: Isolation by distance
*F*_ST_: Fixation index
MAF: Minor allele frequency
CV error: Cross-validation error
LD: Linkage disequilibrium
LFMM: Latent factor mixed models
MYA: Million years ago
GO: Gene ontology
MAPK: Mitogen-activated protein kinase
AMPK: AMP-activated protein kinase
mTOR: Mammalian target of rapamycin
UTRs: Untranslated regions
SFS: Site frequency spectrum
SNMF: Sparse non-negative matrix factorization
KEGG: Kyoto Encyclopedia of Genes and Genomes
