# Supplementary material for: Population genome-wide analysis reveals historical divergence and adaptive signals in Rubroshorea leprosula (Dipterocarpaceae), a near-threatened tropical forest tree
Source: BMC Genomics. 2026 May 29;27:642. doi: 10.1186/s12864-026-12992-5 (PMC13420863; doi:10.1186/s12864-026-12992-5)
Supplement: Supplementary file 1 — Supplementary Fig. S1. Supplementary Fig. S2. Supplementary Fig. S3. Supplementary Table S1. Supplementary Fig. S4. Supplementary Fig. S5. [file 12864_2026_12992_MOESM1_ESM.docx]

**Supplementary Information**


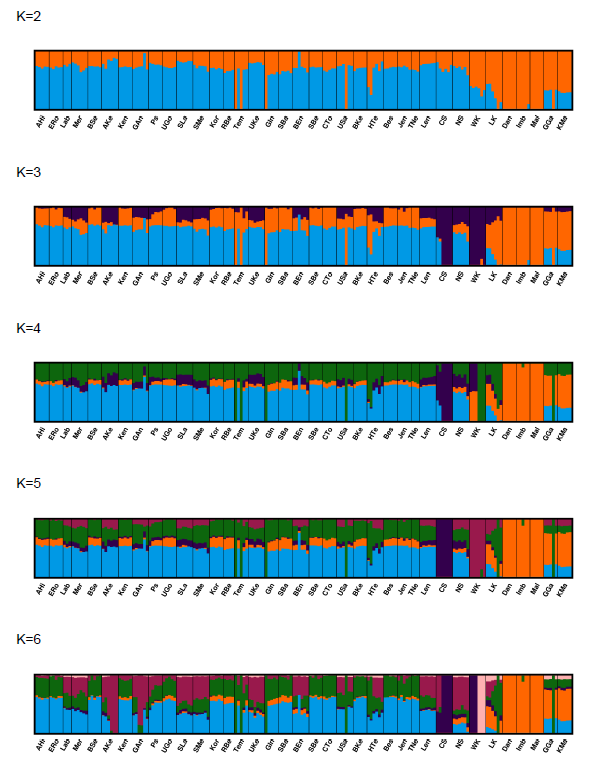


**Peninsular Malaysia**

**Borneo**

**Sumatra**

(A)


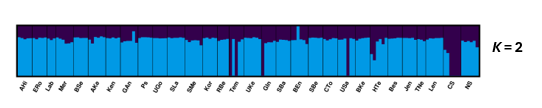


**Western group (Cluster A)**


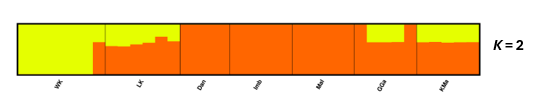


**Eastern group (Cluster B)**

(B)

**Supplementary Fig. S1** Genetic structure of *Rubroshorea leprosula* based on the pruned neutral SNP dataset. **(A)** Global ADMIXTURE analysis at the optimal *K* = 2, identifying two primary lineages. **(B)** Nested ADMIXTURE analysis within each primary lineage. The Western lineage (*K* = 2) differentiates the Sumatra population from Peninsular Malaysia, while the Eastern lineage (*K* = 2) reveals internal sub-structuring and heterogeneity within Borneo.


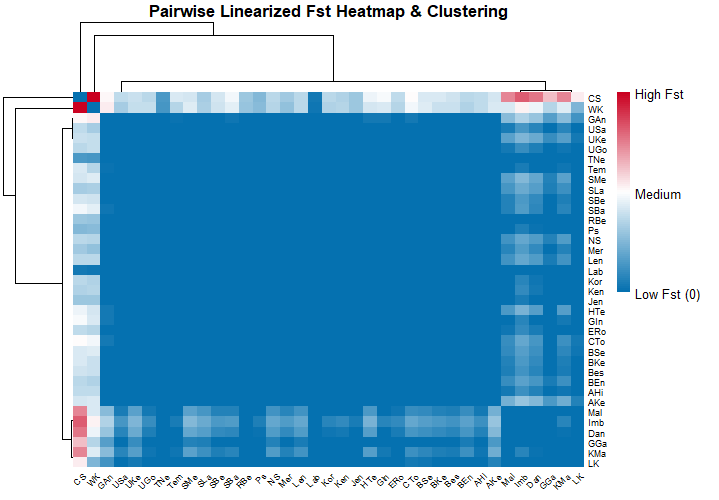


**Supplementary Fig. S2** Heatmap of pairwise *F*_ST_ estimates among 37 populations of *R. leprosula*. Colour intensity represents the degree of genetic differentiation, with values ranging from 0.000 (blue) to 0.075 (red). The dendrogram (top and left) illustrates hierarchical clustering based on genetic similarity.


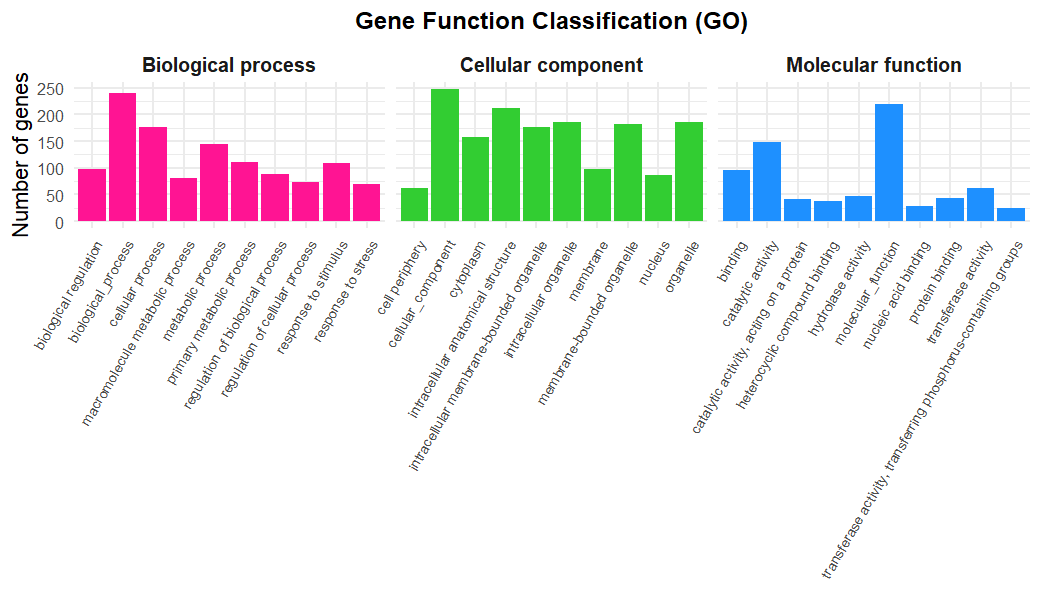

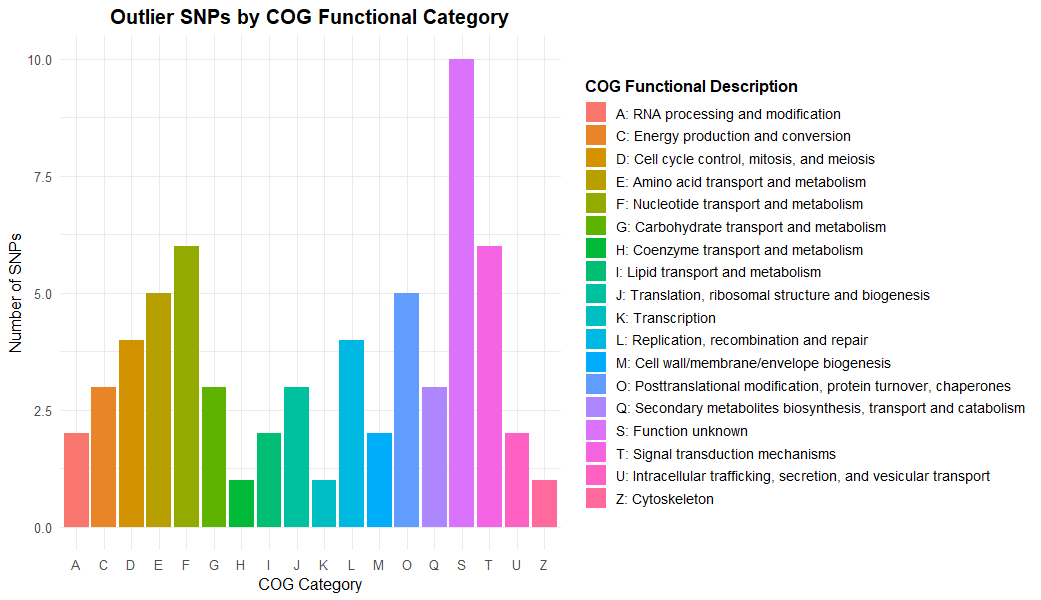


(A)

(B)

(D)

(C)


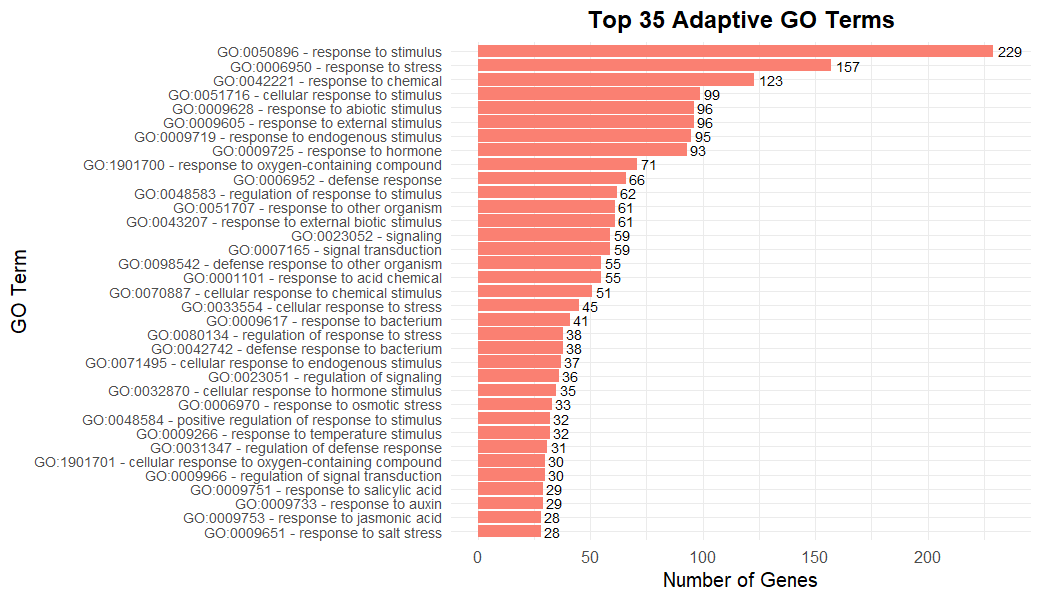


**Top 35 adaptation-related GO terms**


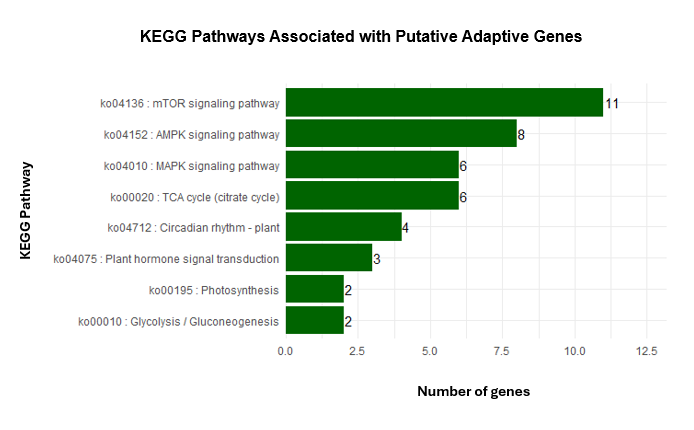


**Supplementary Fig. S3** Functional classification and enrichment of genes associated with pairwise *F*_ST_-based outlier SNPs between Cluster A and Cluster B of *Rubroshorea leprosula.* (A) Distribution of outlier SNPs across Clusters of Orthologous Groups (COG) functional categories. (B) Gene Ontology (GO) classification of candidate genes under Biological Process, Cellular Component and Molecular Function categories. (C) Top 35 significantly enriched GO terms related to putative adaptive functions. D) KEGG pathways associated with putative adaptive genes, highlighting major stress response and regulatory pathways.

**Supplementary Table S1** Climatic variables for the 37 sampled populations of *Rubroshorea leprosula*, including mean maximum temperature of the warmest month (BIO5, °C), mean minimum temperature of the coldest month (BIO6, °C), annual precipitation (BIO13, mm), precipitation of the driest month (BIO14, mm) and elevation (m)

| Population | BIO5 | BIO6 | BIO13 | BIO14 | Elevation |
| --- | --- | --- | --- | --- | --- |
| AHi | 31.648 | 22.174 | 231 | 119 | 26 |
| ERo | 30.486 | 21.497 | 487 | 134 | 203 |
| Lab | 30.107 | 21.111 | 300 | 125 | 274 |
| Mer | 31.482 | 22.425 | 555 | 153 | 28 |
| BSe | 31.521 | 22.019 | 211 | 95 | 82 |
| AKe | 31.858 | 22.206 | 222 | 88 | 32 |
| Ken | 31.388 | 21.302 | 264 | 105 | 206 |
| GAn | 30.575 | 20.828 | 247 | 107 | 329 |
| Ps | 31.734 | 21.868 | 254 | 104 | 121 |
| UGo | 30.895 | 20.892 | 316 | 139 | 257 |
| SLa | 31.266 | 21.268 | 290 | 123 | 209 |
| SMe | 31.683 | 22.201 | 247 | 87 | 26 |
| Kor | 28.704 | 18.210 | 384 | 138 | 695 |
| RBe | 30.929 | 21.075 | 313 | 73 | 316 |
| Tem | 27.981 | 17.442 | 362 | 102 | 867 |
| UKe | 30.196 | 20.086 | 379 | 170 | 382 |
| GIn | 31.721 | 21.477 | 397 | 97 | 247 |
| SBa | 31.681 | 21.065 | 273 | 40 | 227 |
| BEn | 31.820 | 21.372 | 361 | 71 | 197 |
| SBe | 31.070 | 20.613 | 310 | 95 | 248 |
| CTo | 32.113 | 21.878 | 646 | 89 | 81 |
| USa | 31.059 | 21.095 | 597 | 101 | 219 |
| BKe | 31.432 | 21.638 | 674 | 119 | 94 |
| HTe | 30.734 | 20.883 | 672 | 143 | 218 |
| Bes | 31.978 | 22.461 | 669 | 151 | 32 |
| Jen | 31.606 | 21.476 | 291 | 122 | 83 |
| TNe | 30.895 | 20.994 | 275 | 96 | 161 |
| Len | 29.208 | 19.100 | 307 | 125 | 536 |
| CS | 29.981 | 21.373 | 287 | 110 | 215 |
| NS | 21.324 | 10.039 | 271 | 101 | 2043 |
| WK | 27.861 | 18.114 | 405 | 212 | 841 |
| LK | 32.281 | 22.134 | 321 | 114 | 77 |
| Dan | 29.788 | 21.241 | 232 | 145 | 300 |
| Imb | 30.032 | 21.495 | 241 | 169 | 254 |
| Mal | 29.164 | 20.591 | 259 | 155 | 442 |
| GGa | 31.351 | 22.265 | 696 | 143 | 98 |
| KMa | 31.612 | 22.328 | 762 | 178 | 54 |


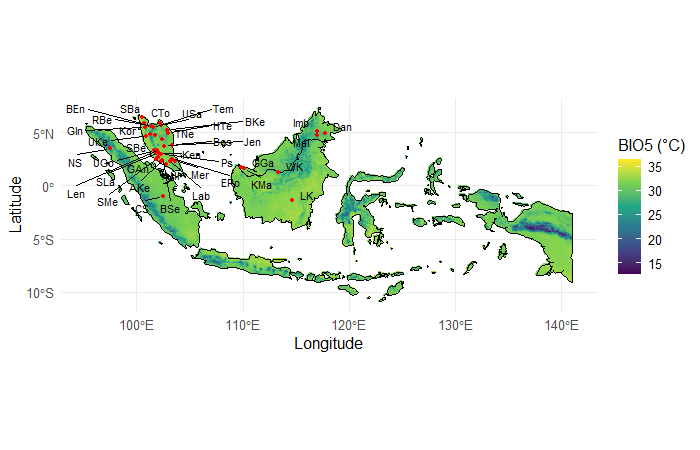


(A)


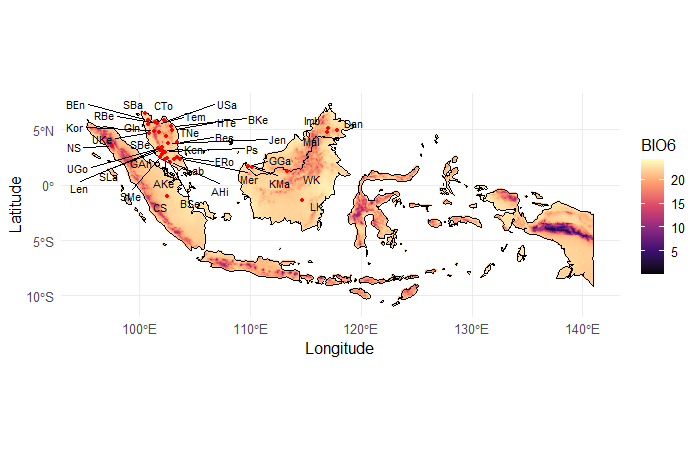


(B)


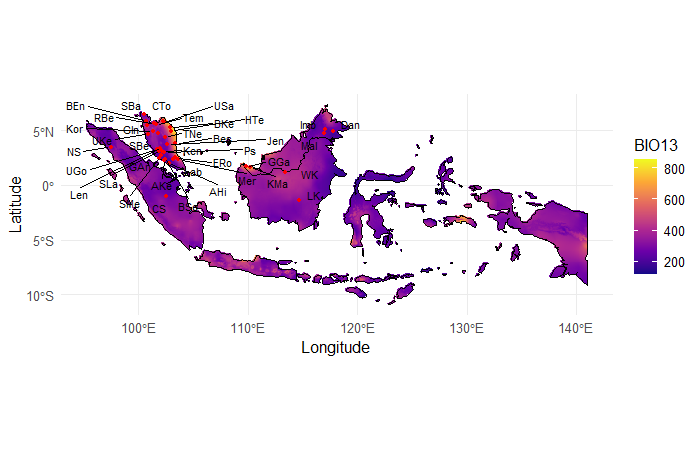


(C)


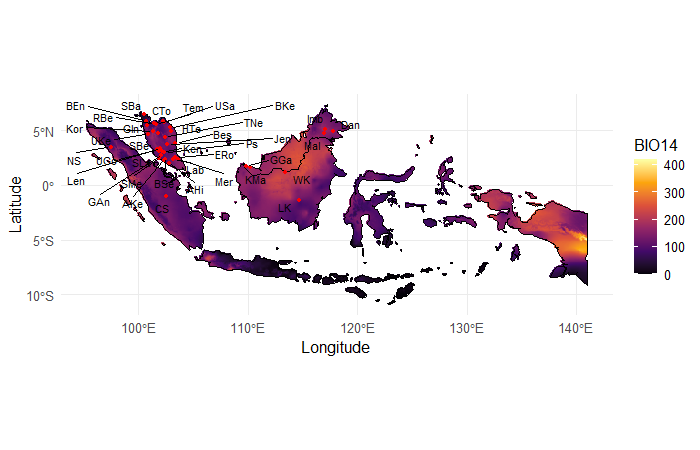


(D)


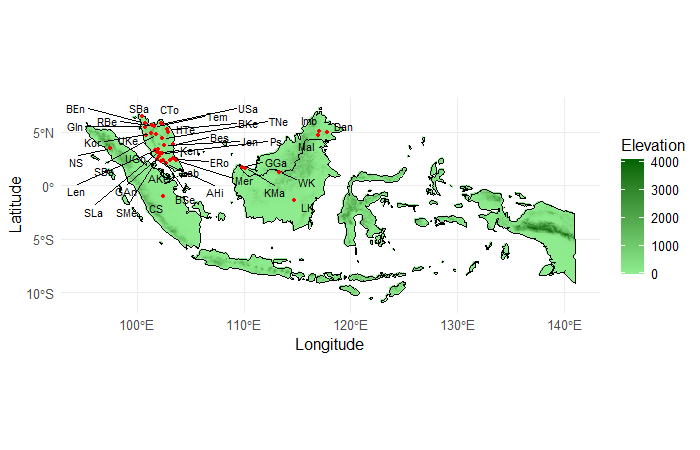


(E)

**Supplementary Fig. S4** Spatial distribution of environmental variables across the 37 sampled populations of *Rubroshorea leprosula*, showing variation in (A) mean maximum temperature of the warmest month (BIO5, °C), (B) mean minimum temperature of the coldest month (BIO6, °C), (C) annual precipitation (BIO13; mm), (D) precipitation of the driest month (BIO14, mm) and (E) elevation


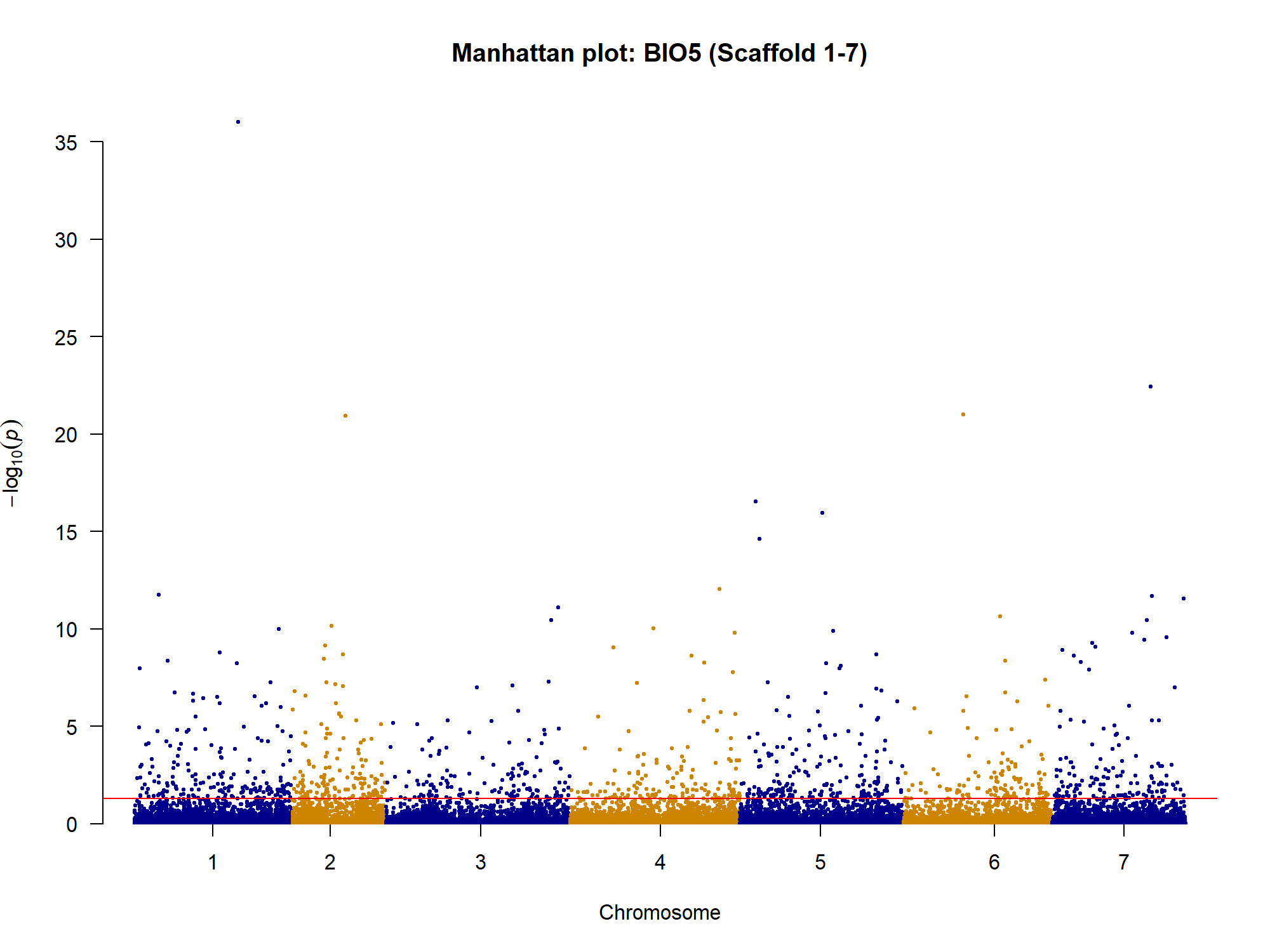


(A)

Scaffold


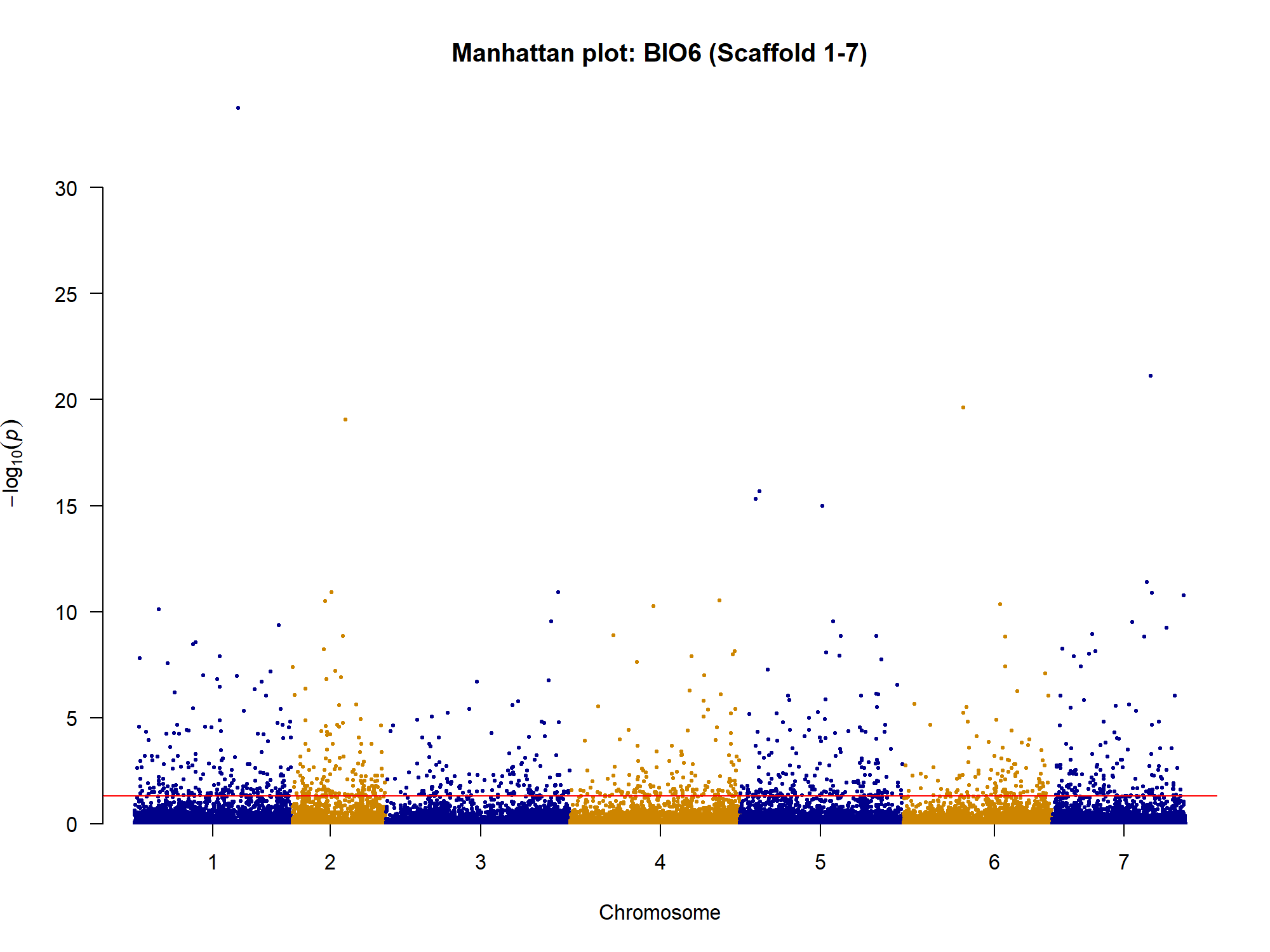


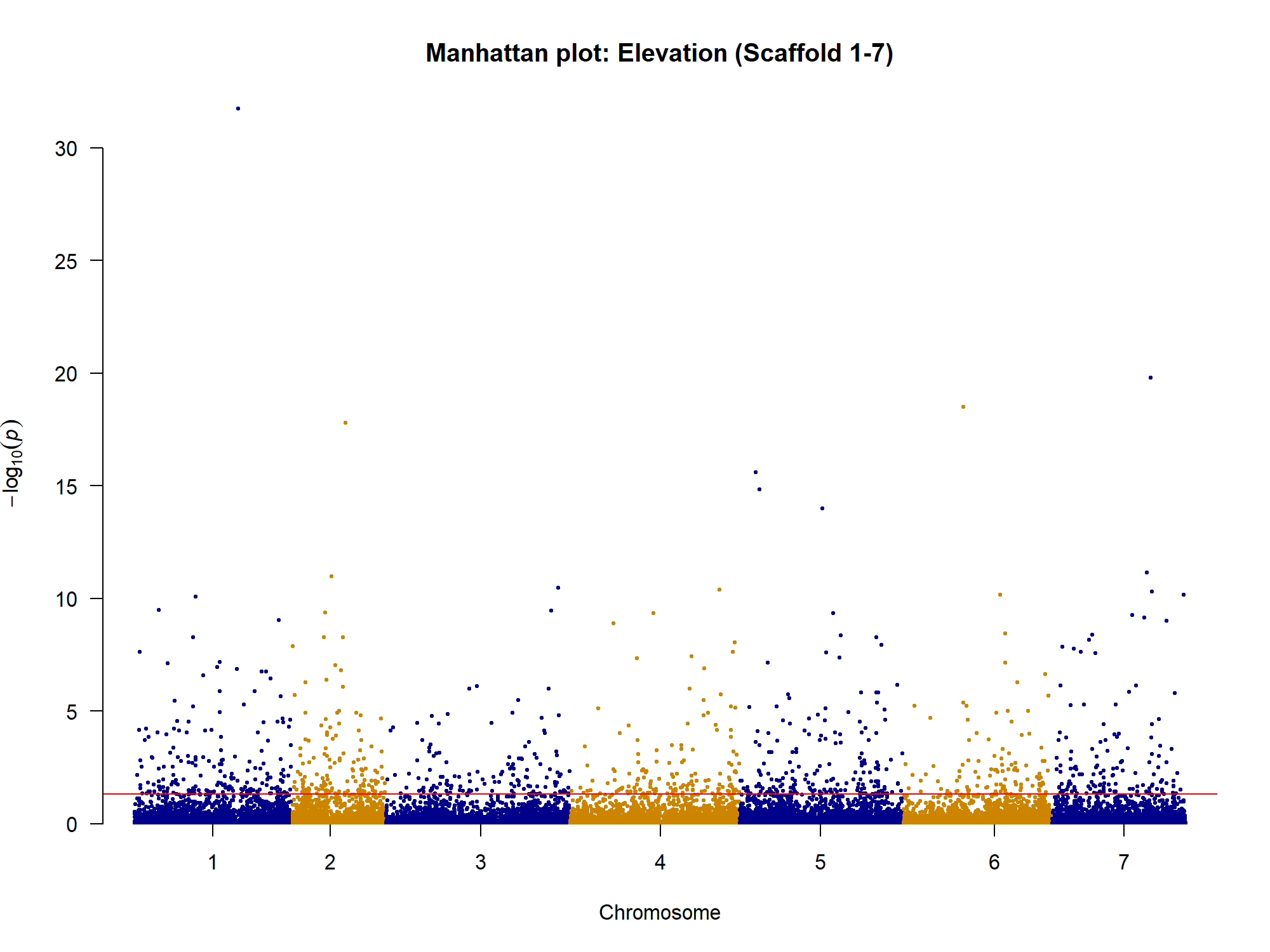


Scaffold

(B)

Scaffold

(C)


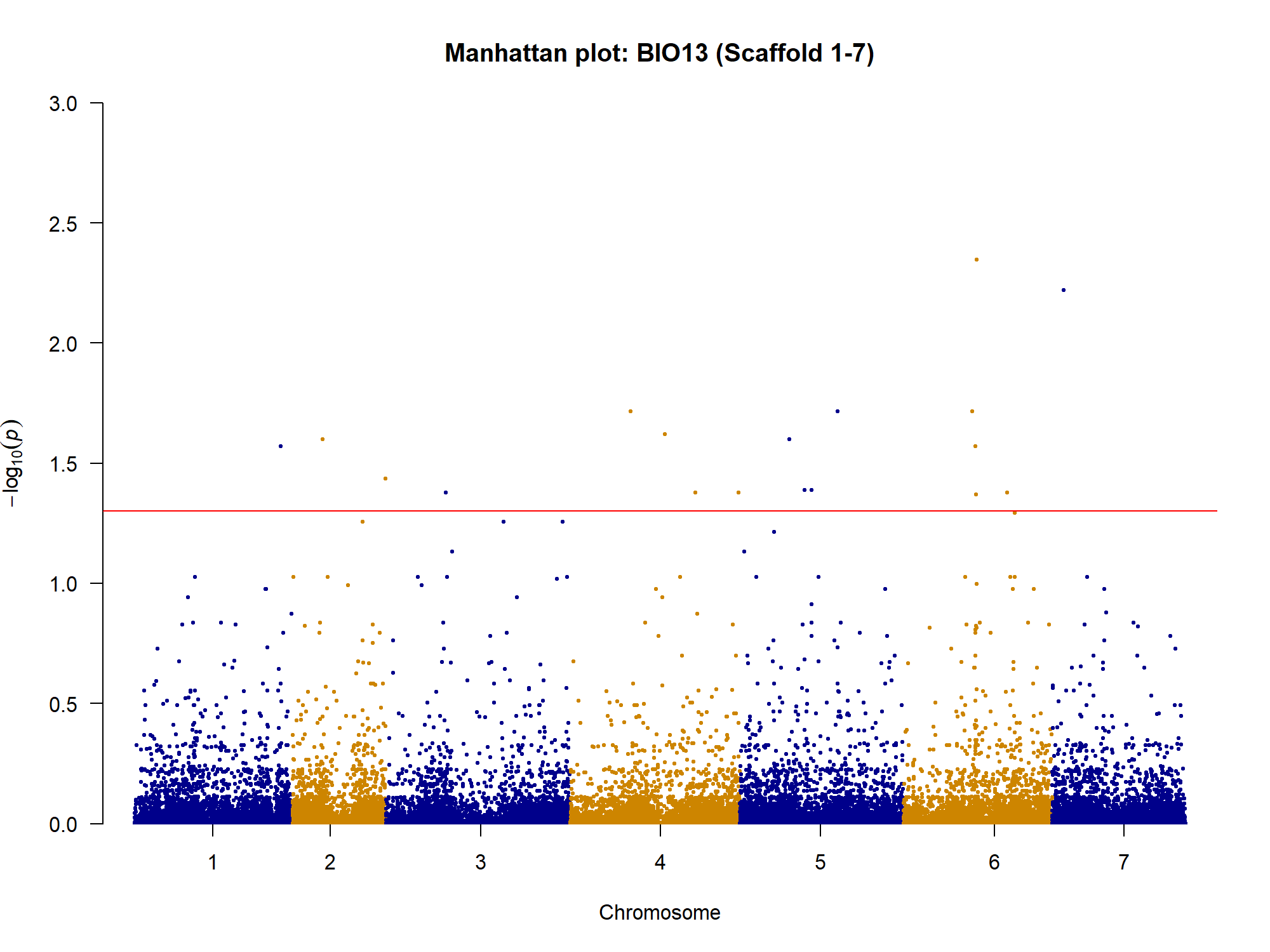


(D)

Scaffold


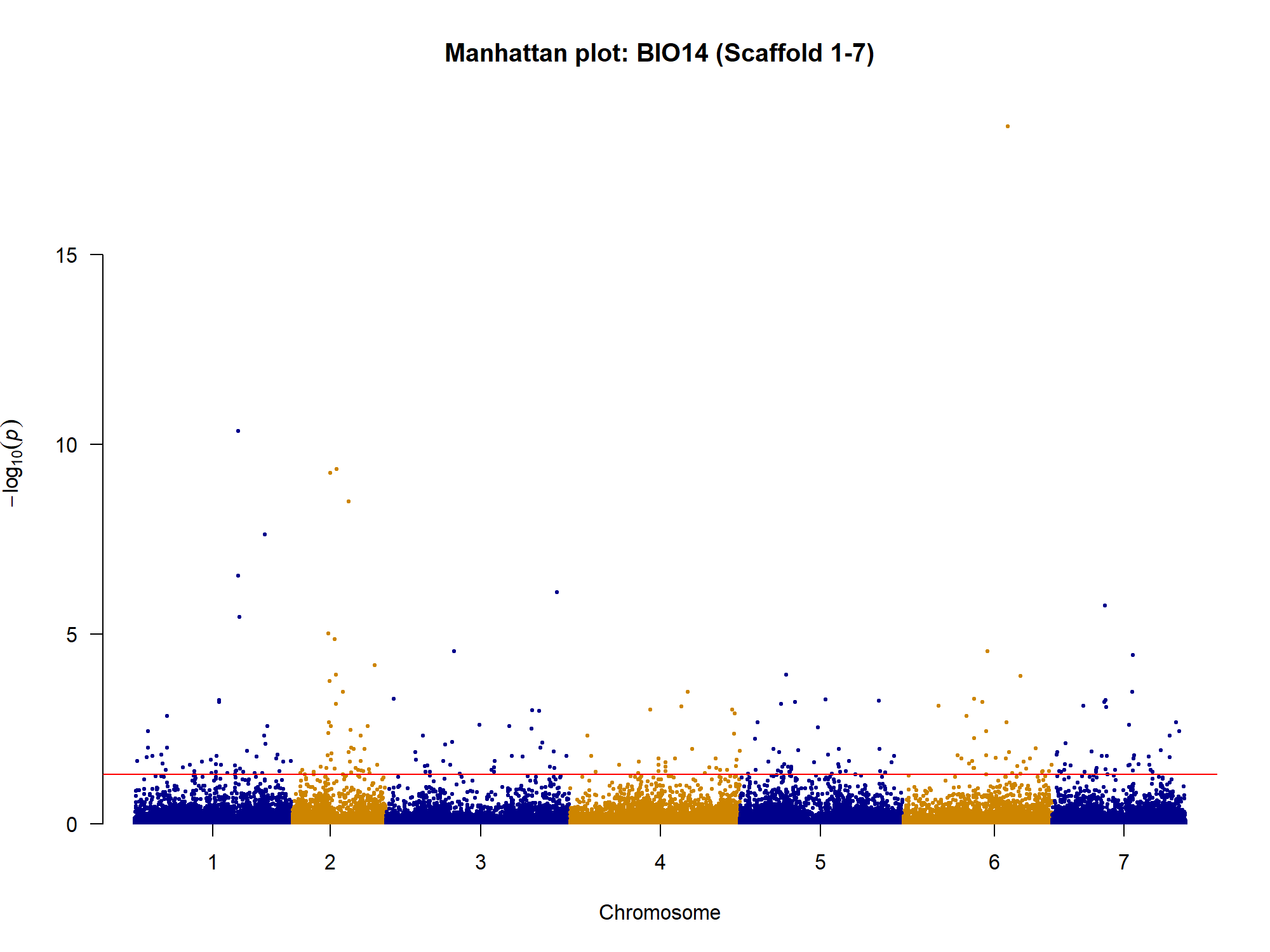


(E)

Scaffold

**Supplementary Fig. S5** Manhattan plots showing genome-wide SNP associations with environmental variables in *Rubroshorea leprosula*: (A) Maximum temperature of warmest month (BIO5), (B) Minimum temperature of coldest month (BIO6), (C) Elevation, (D) Annual precipitation (BIO13) and (D) Precipitation of driest month (BIO14). The red horizontal line indicates the Benjamini-Hochberg significance threshold (adjusted *p* < 0.05). SNPs exceeding this threshold are considered significantly associated with the corresponding environmental variable.
